# Hyperelastic Cardiovascular NN–FE: A Framework for Integrating Nonlinear Finite Element Solvers with Neural Networks for the Hyperelastic Modeling of Cardiac Valve Tissue

**DOI:** 10.21203/rs.3.rs-11002082/v1

**Published:** 2026-09-14

**Authors:** Maedeh Makki, Gerhard A. Holzapfel, Maziar Raissi, Chung-Hao Lee

**Affiliations:** 1 Biomechanics and Biomaterials Design Laboratory (BBDL), Department of Bioengineering, The University of California, Riverside, Riverside, CA 92521, USA; 2 Institute of Biomechanics, Graz University of Technology, Austria; 3 Department of Structural Engineering, Norwegian University of Science and Technology, Trondheim, Norway; 4 Department of Mathematics, The University of California, Riverside, Riverside, CA 92521, USA

**Keywords:** Atrioventricular heart valves, biaxial loading test, finite element analysis, physics-informed neural networks (PINNs), real-time neural network adaptation, solver integration

## Abstract

Accurately predicting tissue mechanics of cardiac valves is crucial for computational biomechanics and surgical planning. Conventional finite elements (FEs) entail high computational costs when modeling real-world applications, whereas purely data-driven approaches lack physical consistency and interpretability. We present a hybrid, physics-informed neural network framework called NN–FE, which combines a feedforward neural network with a nonlinear FE solver to predict the deformation of the tricuspid valve posterior leaflet (TVPL) under quasi-static biaxial loading. For collection of biomechanical data, TVPL tissues were subjected to seven biaxial loading ratios. The tissue response was characterized using a Fung-type hyperelastic model with parameters identified via differential evolution optimization. A six-layer feedforward neural network, comprising five hidden layers and one output layer, predicts pointwise displacements as functions of spatial coordinates, time, and loading ratio. These predictions serve as the trial fields for the FE solver, which enforces equilibrium through Newton-Raphson iterations. The FE-corrected displacement fields provide physics-consistent training targets, enabling the neural network to learn the constitutive behavior without explicit partial differential equation residual terms or empirical loss weighting. Model performance was evaluated using leave-one-ratio-out cross-validation across six case studies. We showed that the proposed framework achieved mean absolute percentage errors (MAPE) less than 15% for displacement predictions and temporal coefficients of determination exceeding 0.85 when predicting out-of-distribution scenarios. Additionally, tissue stretch predictions demonstrated superior accuracy (MAPE<1.4%), while stress predictions exhibited a MAPE of less than 7.8%. The NN–FE framework represents a computationally efficient surrogate model suitable for real-time applications in cardiovascular biomechanics.

## 1. Introduction

Understanding and predicting large deformations of soft biological tissues is of fundamental importance for numerous biomedical applications—ranging from surgical simulation and medical device development to image-guided interventions and mechanobiological studies. Soft biological tissues show anisotropic and highly nonlinear behavior under large strains [1], and hyperelastic material models are frequently employed to describe their mechanical response under various loading conditions [2]. For example, the behavior of cardiac valve tissue under quasi-static loading conditions has been simulated using an incompressible hyperelastic material model [3,4].

Classical constitutive models, such as the neo-Hookean, Mooney–Rivlin, Ogden and Holzapfel-type models, provide versatile strain-energy functionals capable of capturing the nonlinear elastic behavior of materials [5]. Unlike linear infinitesimal deformation theory, the nonlinear constitutive relation between stress and strain which is based on finite deformation theory [6], makes solving boundary-value problems computationally expensive [7]. This limits high-fidelity hyperelastic models in real-time scenarios and motivates the development of alternative numerical techniques for solving nonlinear material models [8].

Advances in data-driven modeling and computational solvers have enabled neural networks to solve nonlinear physical systems [9,10]. In computational mechanics, this has motivated frameworks that replace the traditional constitutive laws with supervised learning to solve nonlinear elastic boundary-value problems [11,12]. These methods allow material responses to be captured directly from the data rather than through empirical constitutive equations. Standard artificial neural networks (ANNs) used for constitutive modeling lack inherent physical constraints, leading to instabilities and nonphysical behavior [13,14]. To address the limitations of purely data-driven models, physics-informed neural networks (PINNs) [15,16] were developed. These solve inverse problems by incorporating partial differential equations (PDEs) and constitutive constraints into the training objective functions, thereby ensuring physical consistency—even with limited data—through automatic differentiation [17].

Early applications of PINNs in the field of solid mechanics demonstrated their fundamental potential: Haghighat *et al*. [18] developed an approach for linear elasticity and nonlinear elastoplasticity [18], while Zhang *et al*. [19,20] identified nonhomogeneous hyperelastic materials as well as internal defects [19,20]. Although these initial PINN models were based on the strong form of the PDEs, high-order derivatives often led to computational bottlenecks in cases of large strain regimes. To address this issue, Samaniego *et al*. [21] and Nguyen-Thanh *et al*. [12] proposed the “deep energy method” for hyperelasticity [21,22]. By minimizing the potential energy functional instead of the partial differential equation residual, the deep energy method bypasses mesh discretization and offers superior stability.

Subsequent algorithmic improvements have refined both variational and differential approaches for complex materials. For example, Fuhg and Bouklas [23] introduced the mixed deep energy method, which incorporates stress outputs to resolve stress concentration that are often not captured by the conventional deep energy method. Conversely, to stabilize the strong-form approach during extreme stretching, Luo *et al*. [24] developed a stepwise PINN strategy that solves large deformation problems through sequential training. These methodological refinements, alongside recent work by Song and Jin [25] on identifying the parameters of the Arruda-Boyce model from noisy data and Zhu *et al*. [26] on transfer learning for parameter identification in soft materials, have significantly expanded the robustness of physics-aware learning in soft tissue mechanics. These methodological advancements have enabled high-fidelity clinical translation in cardiovascular mechanics. Frameworks have been developed to estimate patient-specific myocardial properties and motion tracking from clinical imaging [27,28], as well as to map cardiac electrophysiology and tissue heterogeneities [29,30]. Importantly, the ADEPT framework [31] enables the determination of the elastic properties of valve tissue directly from 3D echocardiography. Despite these advances, the “stiff” equations characteristic of hyperelastic tissue continue to pose challenge for training the neural network model, particularly regarding spectral bias and non-uniform convergence rates [32].

Given the limitations of per-instance solvers like PINNs, researchers have increasingly turned to neural operators that learn the underlying solution mapping across entire function spaces. The Fourier neural operator (FNO), introduced by Li *et al*. [33] has emerged as a cornerstone of this paradigm, offering resolution-invariant inference and significant speedups by operating in the frequency domain. These methodological developments are particularly transformative for biomechanics; e.g., Geo-FNO was developed to accommodate the irregular, patient-specific geometries inherent in cardiac and vascular modeling [34]. Beyond geometry, physics-guided FNOs have been successfully applied to characterize complex biological tissues, such as porcine tricuspid valve leaflets, by predicting displacement fields directly from digital image correlation measurements [35]. However, comprehensive demonstrations for anisotropic hyperelastic soft tissues under repeated biaxial loading protocols remain limited.

In this study, we present a hybrid, physics-informed neural network (NN) framework—referred to as NN-FE—that combines a feedforward neural network with a nonlinear FE solver to predict the anisotropic hyperelastic response of a representative biological tissue: TVPL under quasi-static biaxial loading. The proposed NN–FE framework employs a “solver-in-the-loop approach” in which the neural network generates pointwise displacement predictions that are subsequently refined through FE analysis in Abaqus to ensure satisfaction of the equilibrium equation. Unlike the conventional PINNs, the proposed method is not mesh-free, as equilibrium is enforced through FE discretization; however, this design avoids the convergence issues commonly reported for PINNs in strongly nonlinear hyperelastic problems [32]. Moreover, since the governing physics is enforced directly by the nonlinear FE solver, the loss function contains a single residual term, eliminating the need to balance multiple physics-based residuals through empirical weighting strategies. To generate the experimental data for calibrating and testing our NN–FE framework, the porcine TVPL specimen is subjected to displacement-controlled biaxial experiments at seven loading ratios $\left(F_{X}:F_{Y}=1:1,1:0.75,1:0.5,1:0.25,0.75:1,0.5:1,0.25:1\right)$ following preconditioning protocols (where $F_{X}$ and $F_{Y}$ denote the reaction forces in the X- and Y-directions). The tissue response is then characterized by using a Fung-type hyperelastic constitutive model, with material parameters identified through differential evolution optimization incorporating a Weibull-flight mutation operator. The proposed neural network, consisting of six hidden layers with 128 neurons each and hyperbolic tangent activation functions, is trained using mini-batch gradient descent with a physics-based loss that minimizes the discrepancy between the NN–FE predictions and FE-corrected displacement fields. This coupled approach enables the network to learn displacement fields consistent with the governing equations of continuum mechanics while maintaining excellent computational efficiency for potential real-time applications in cardiovascular bioengineering. The remainder of the manuscript is organized as follows: Section 2 presents the governing equations and the framework for modeling hyperelastic materials. Section 3 introduces the proposed hybrid neural network–finite element (NN–FE) framework, followed by the experimental data acquisition described in Section 4. Section 5 reports the results and validation studies. The key findings of numerical studies and the study’s limitations are discussed in Section 6. Concluding remarks are provided in Section 7.

## 2. Methods

This study aims to predict how the tricuspid valve posterior leaflet (TVPL) specimens deform under quasi-static biaxial loading conditions. Here, the TVPL specimens were assumed to be anisotropic hyperelastic solids, which is consistent with previous experimental and constitutive modeling studies [27, 29]. Although the composition and microstructure of the valve leaflets [30,36]—comprising collagen, elastin, and glycosaminoglycans—are known to contribute to the viscoelastic behavior [31], experimental results from stress-relaxation and creep tests reported in previous studies showed that, under low strain-rate loading, i.e., quasi-static conditions typical of many biomedical applications, the valvular tissue can withstand loading without noticeable time-dependent (i.e., viscous) effects, and its stress-strain response can be reasonably represented by time-independent (pseudoelastic) hyperelastic models [37].

In the following subsections, we provide a brief overview of the hyperelastic constitutive model used to model the mechanical response of heart valve tissue; this model was tested under seven biaxial loading ratios $\left(F_{x}:F_{y}\right)$ covering a wide range of mechanical tissue deformations.

### 2.1 Kinematics and hyperelastic constitutive relationships

**Figure 1 –:**
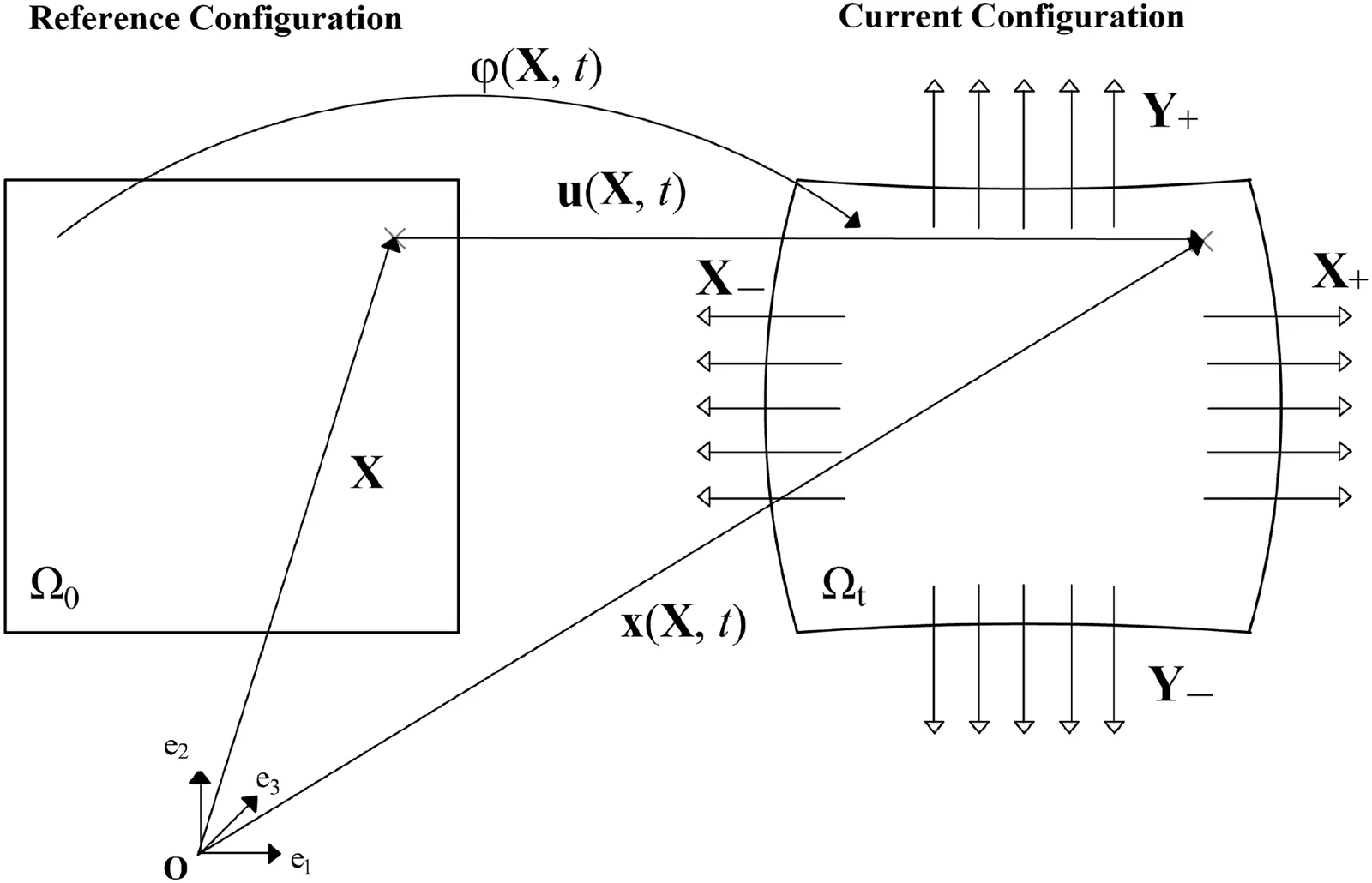
Illustration of the motion of a body from its reference configuration $\Omega_{0}$ to its current configuration $\Omega_{t}$ using the mapping 𝛗(X,t).

In the context of continuum mechanics [6,7,38], the deformation of a material body is described using a smooth, time-dependent mapping $\varphi :\Omega_{0}\times [0,T]\to R^{3}$ (Fig. 1), where $\Omega_{0}$ denotes the reference configuration. Under this motion, each material point $X\in \Omega_{0}$ is mapped to its spatial position x=φ(X,t) in the current configuration $\Omega_{t}$. The corresponding displacement field is given by u(X,t)=φ(X,t)-X. To characterize the local deformation, the deformation gradient F=∂x/∂X is employed, which captures the stretching and rotation of infinitesimal line elements, while the associated Jacobian J=det(F)>0 provides a measure of the local volume change. The right Cauchy-Green tensor $C=F^{\text{T}}F$ is used to isolate mechanical strain from rigid-body motions, and the Green-Lagrange strain tensor is determined by $E=\frac{1}{2}(C-I)$ where I is the second-order unit tensor.

The mechanical behavior of hyperelastic materials is characterized by stress measures derived from the strain-energy function ψ [39, 40]. The Cauchy stress tensor 𝛔 represents the true stress, defined as force per unit area in the deformed (spatial) configuration. For hyperelastic materials with a given strain-energy function ψ, the Cauchy stress tensor and the second Piola-Kirchhoff (PK) stress tensor are given by

(1) $$𝛔=\frac{1}{J}{FSF}^{T},\;S=\frac{\partial \psi }{\partial E}=2\frac{\partial \psi }{\partial C},$$

On the other hand, the first PK stress tensor P is relating forces to surface areas in the undeformed configuration, i.e.

(2) P=FS.

In contrast to the Cauchy stress tensor, the first PK stress tensor P is generally unsymmetric $\left({PF}^{T}={FP}^{T}\right)$ and is commonly referred to as the nominal stress tensor in experimental context. The two stress measures are related via the Piola transformation, $P=J\;𝛔F^{-T}$, which maps the spatial stress 𝛔 to its corresponding material form P [6].

### 2.2 Fung-type hyperelastic model

In this study, the Fung model was used to describe the hyperelastic behavior, as this model is frequently used to model the highly nonlinear, exponential stiffening of collagen fibers in soft tissue under large deformations [41, 42]. The strain-energy function ψ for the generalized anisotropic Fung model is given by

(3) $$\psi \left(\bar{E},J\right)=\psi_{\text{iso}}+\psi_{\text{vol}},\;\psi_{\text{iso}}=\frac{c}{2}\left[\text{exp}\left(Q\right)-1\right],\;\psi_{\text{vol}}=\kappa \left(\frac{J^{2}-1}{2}-\text{ln}\;J\right),$$

where $\psi_{\text{iso}}$ represents the isochoric response, governed by the parameter c, and $\psi_{\text{vol}}$ describes the strain energy associated with volumetric changes, with κ serving as a penalty parameter to enforce material incompressibility [6, 43]. The exponent Q is defined as

(4) $$Q=\bar{E}:b:\bar{E}={\overset{‾}{E}}_{ij}b_{\text{ijkl}}{\overset{‾}{E}}_{kl},$$

where $\bar{E}=(\bar{C}-I)/2$ denotes the isochoric (modified) Green-Lagrange strain tensor, $\bar{C}=J^{-2/3}C$ is the modified right Cauchy-Green tensor, and $[b]=\left\{b_{\text{ijkl}}\right\}$ represents a dimensionless, symmetric fourth-order tensor of material parameters characterize the anisotropic response of the soft tissue. The second PK stress tensor can then be derived as follows:

(5) $$S=\frac{\partial \psi }{\partial E}=S_{\text{iso}}+S_{\text{vol}},\;S_{\text{iso}}=\frac{\partial \psi }{\partial \bar{E}}:\frac{\partial \bar{E}}{\partial E}=J^{-2/3}\left[T-\frac{1}{3}(T:C)C^{-1}\right]\;S_{\text{vol}}=\frac{\partial \psi }{\partial J}\frac{\partial J}{\partial E}=\kappa J(J-1)C^{-1},$$

where

(6) $$T\equiv \frac{\partial \psi_{\text{iso}}}{\partial \bar{E}}=c\;\text{exp}\left(Q\right)\;b:\bar{E}.$$

### 2.3 Simplified relationships due to planar biaxial loading

Assuming shear-free, nearly incompressible materials, both the isochoric strain tensor $\bar{E}$ and the second PK stress tensor S remain diagonal in the principal basis $\left(e_{1},e_{2},e_{3}\right)$, since only the normal strain components contribute to them. This simplification arises directly from the classical theory of hyperelasticity when deformation occurs along the principal directions [39]. The expression for the general scalar exponent Q, as defined in Eq. (4), can be rewritten in Voigt notation as follows:

(7) $$Q={𝛜}^{T}b𝛜,$$

where 𝛜 is the 6 × 1 strain vector, and b is a symmetric 6 × 6 matrix containing the anisotropic material parameters. Assuming vanishing (or negligible) shear components (${\overset{‾}{E}}_{ij}=0$ for i≠j), the vector 𝛜 reduces to its first three normal strain components:

(8) $$𝛜={\left[{\overset{‾}{E}}_{11},{\overset{‾}{E}}_{22},{\overset{‾}{E}}_{33}\right]}^{\text{T}},$$

leading to the following reduced scalar form:

(9) $$Q=\sum_{i=1}^{3}\sum_{j=1}^{3}b_{\text{iijj}}{\overset{‾}{E}}_{ii}{\overset{‾}{E}}_{jj}\;=b_{1111}{\overset{‾}{E}}_{11}^{2}+b_{2222}{\overset{‾}{E}}_{22}^{2}+b_{3333}{\overset{‾}{E}}_{33}^{2}+2b_{1122}{\overset{‾}{E}}_{11}{\overset{‾}{E}}_{22}+2b_{1133}{\overset{‾}{E}}_{11}{\overset{‾}{E}}_{33}+2b_{2233}{\overset{‾}{E}}_{22}{\overset{‾}{E}}_{33}.$$

Using the isochoric–volumetric split in Eq. (5), the normal stress components of S can be computed by

(10) $$S_{ii}=J^{-2/3}\left[T_{i}-\frac{1}{3}\left(\sum_{k=1}^{3}T_{k}C_{kk}\right){C_{ii}}^{-1}\right]+\kappa \;J\left(J-1\right){C_{ii}}^{-1},\;i=1,2,3.$$

Here, the fictitious stress $T_{i}$, derived from the tensor T defined in Eq. (6), is calculated as follows:

(11) $$T_{i}\equiv \frac{\partial \psi_{\text{iso}}}{\partial {\overset{‾}{E}}_{ii}}=c\;\text{exp}(Q)\sum_{j=1}^{3}b_{\text{iijj}}{\overset{‾}{E}}_{jj}.$$

As a consequence, the normal components of the nominal stress tensor $P_{ii}$ are determined by:

(12) $$P_{ii}=\lambda_{i}S_{ii}.$$

These components are expressed in terms of the principal stretches $\lambda_{i}$, which represent the ratios of the deformed to the undeformed lengths along the principal axes. In this coordinate system, the stretches correspond to the diagonal components of the deformation gradient, i.e., $F=\text{diag}\left(\lambda_{1},\lambda_{2},\lambda_{3}\right)$. The resulting in-plane normal stresses $P_{11}$ and $P_{22}$ are then expressed as follows

(13) $$P_{11}=\lambda_{1}S_{11},\;P_{22}=\lambda_{2}S_{22}.$$

In this context, the traction-free boundary condition $\left(S_{33}=0\right)$ was applied in the out-of-plane direction which allows the transmural stretch $\lambda_{3}$ (i.e., in the thickness direction) to be determined analytically using the incompressible constraint J=1.

## 3. Physics-Informed Deep Learning Framework

### 3.1 Neural network architecture for pointwise displacement approximation

Let $\Omega_{0}\subset R^{2}$ be the reference configuration of the tissue material under planar biaxial loading. For a loading state (t,α)—where t denotes the time and α the prescribed biaxial loads (i.e., $F_{X}:F_{Y}$)—the displacement field is approximated by a feedforward neural network $N_{\theta }:R^{4}\to R^{2}$ as

(14) $$\hat{u}\left(X,t,\alpha \right)=N_{\theta }\left(X,t,\alpha \right),\;X=(X,Y{)}^{T}\in \Omega_{0}.$$

**Figure 2 –:**
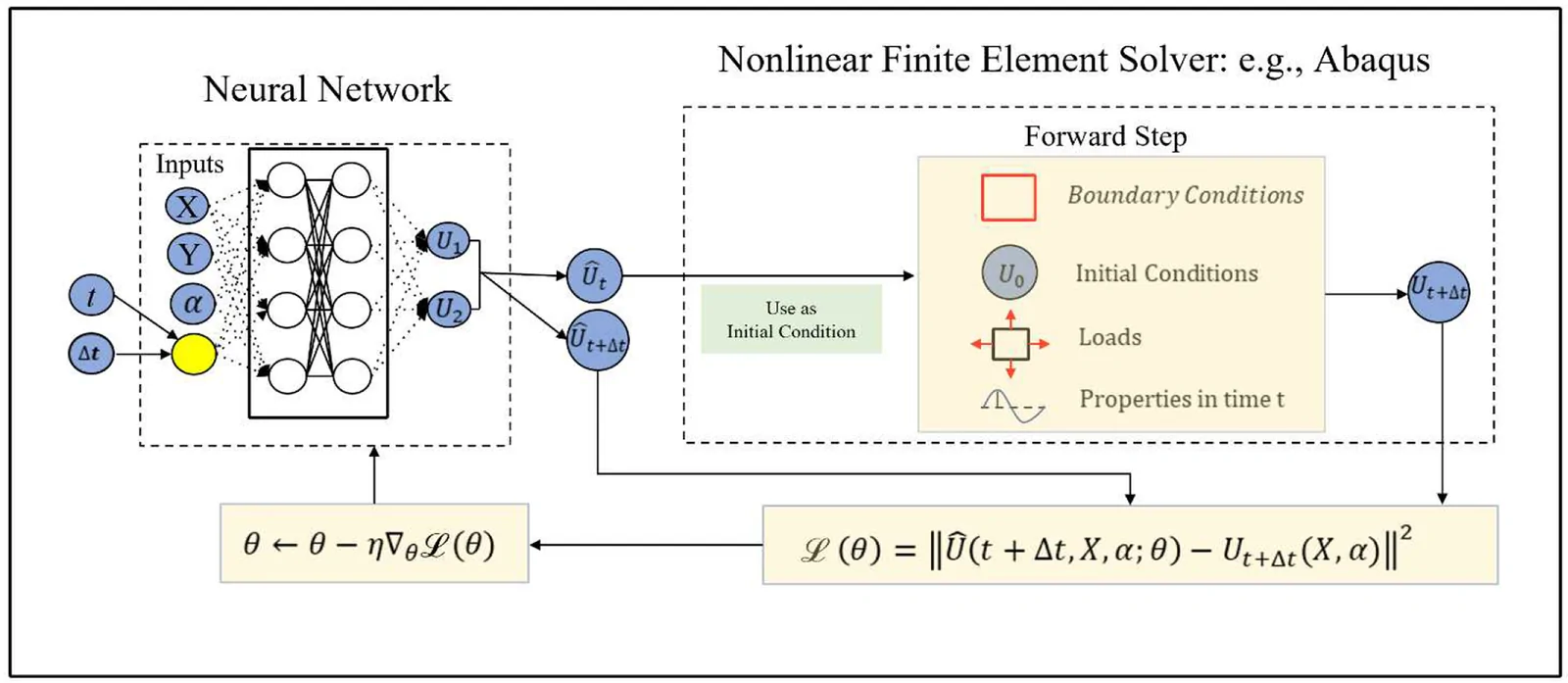
Schematic of the proposed NN–FE applied to modeling the hyperelasticity of a representative tricuspid valve posterior leaflet tissue.

**Figure 3 –:**
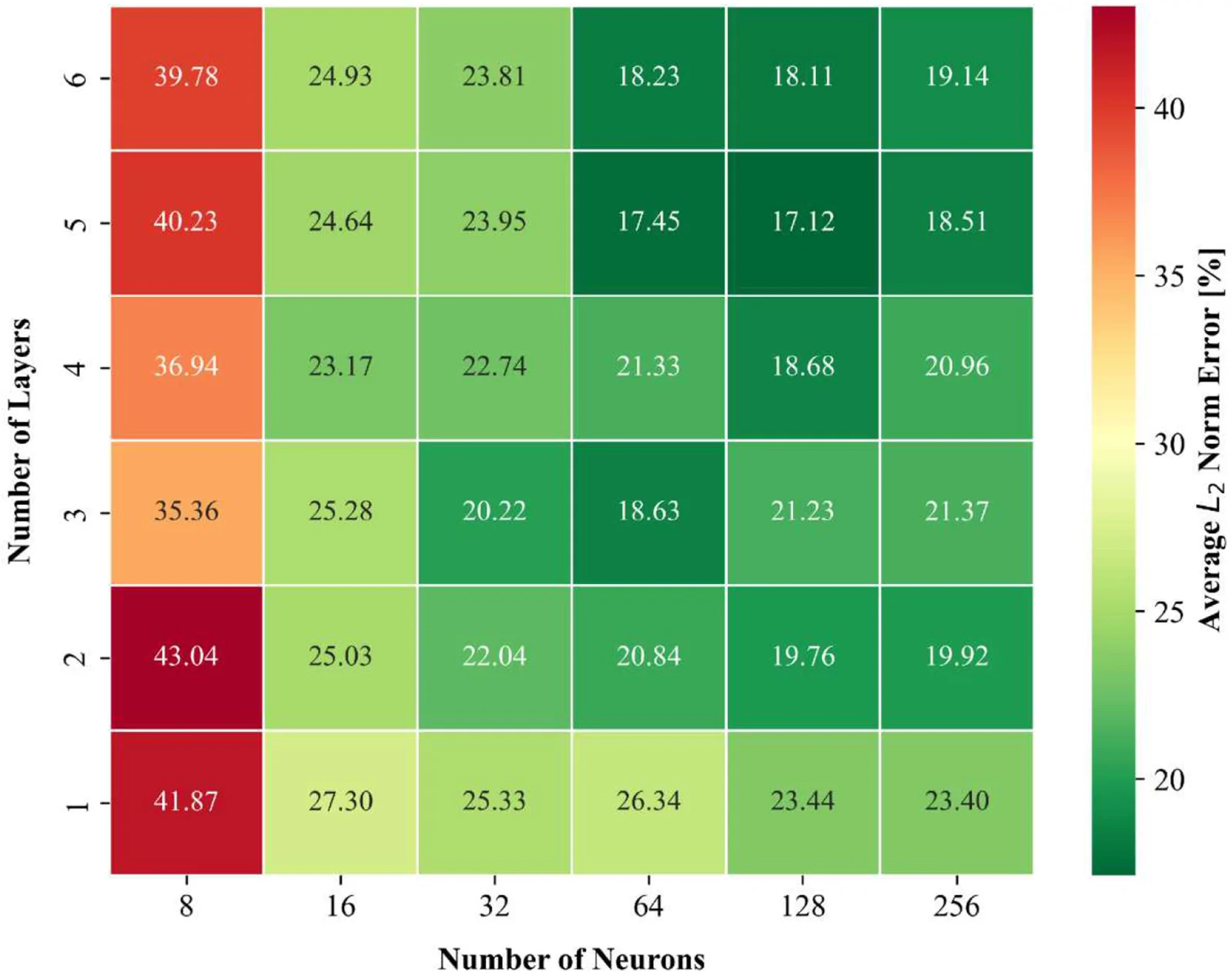
Heatmap of the average displacement error (L_2_ norm, in %) obtained from the hyperparameter tuning study regarding network depth (1 to 6 hidden layers) and width (8 to 256 neurons per layer), used to select the optimal architecture (5 hidden layers, 128 neurons).

The neural network consists of five fully connected hidden layers, each with 128 neurons (Fig. 2). The hyperbolic tangent (tanh) activation function is employed in the hidden layers, whereas the output layer is linear and yields the two displacement components $U_{1}$ and $U_{2}$. This specific architecture was selected based on a comprehensive hyperparameter optimization study in which the network’s depth and width were evaluated in terms of the average ${\text{L}}_{2}$-norm error (Fig. 3). Formally, the mapping performed by the network can be described by the following relationships:

(15) $$h^{0}=[X,Y,t,\alpha {]}^{T},\;h^{l}=\text{tanh}\left(W^{l}h^{l-1}+b^{l}\right),\;l=1,\ldots ,5\;\overset{ˆ}{u}=W^{6}h^{5}+b^{6},$$

where $W^{l}$ and $b^{l}$ denote the trainable weights and biases at layer l. The same network parameters $\theta ={\left\{W^{l},b^{l}\right\}}_{l=1}^{6}$ are shared across all spatial locations (X,Y). Training is performed using the ADAM optimizer with a cosine annealing learning rate schedule varying between 10^−7^ and 10^−3^, following [44]. Different from the original PINNs model, the physical constraints, including the equilibrium, compatibility, and boundary conditions, are not enforced directly at the neural network level but are imposed through the embedded FE operator that will be described in Section 3.3.

### 3.2 Governing equations of quasi-static deformation

The mechanical response of a solid body to external stimuli is governed by the fundamental physical laws. For quasi-static deformations, inertial effects are negligible, and the equilibrium configuration is determined by the balance of linear momentum with the strong form of equilibrium in the material (Lagrangian) description, given by

(16) $$\nabla_{X}\cdot P+f_{b}=0,\;X\in \Omega_{0},\;u=\underline{u},\;X\in \Gamma_{u},$$

where $f_{b}$ is the body force vector, $\nabla_{X}$ · is the divergence operator with respect to the material coordinates X in the reference configuration, $\underline{u}$ is the Dirichlet boundary condition prescribed on the boundary $\Gamma_{u}$, and 0 denotes the zero vector.

### 3.3 Hybrid NN-FE integration

The proposed hybrid NN-FE framework couples a neural network surrogate with a nonlinear FE solver to enforce the governing equations of quasi-static hyperelasticity at the field level (Fig. 2). Training was performed using mini batches composed of $N_{\text{batch}}=10$ independently sampled loading states. At each training iteration, each batch element $b=1,\ldots ,N_{\text{batch}}$ was generated through conditional sampling: the loading ratio $\alpha^{\left(b\right)}$ was first sampled from the discrete set of the experimentally tested ratios $\Gamma =\left\{F_{x}\right.\left.:F_{y}=1:1,1:0.75,1:0.5,1:0.25,0.75:1,0.5:1,0.25:1\right\}$, and the loading step $t^{\left(b\right)}$ was subsequently sampled from the duration of the biaxial experiment conducted at the selected ratio, denoted by the set of admissible time steps. Each batch element is thus uniquely defined by the pair $\left(t^{\left(b\right)},\alpha^{\left(b\right)}\right)$ and was processed independently within the iteration.

For a given batch element $\left(t^{\left(b\right)},\alpha^{\left(b\right)}\right)$, the neural network was evaluated at all nodal positions of the FE mesh. Let ${\left\{X_{i}\right\}}_{i=1}^{N_{\text{nodes}}}$, with $X_{i}={\left(X_{i},Y_{i}\right)}^{T}$, denote the reference coordinates of the mesh nodes ($N_{\text{nodes}}=930$ is the number of FE nodes). The input for the neural network was formed by concatenating the nodal coordinates with the sampled loading parameters, resulting in the following matrix:

(17) $$Z^{\left(b\right)}\in R^{N_{\text{nodes}}\times 4}=\left[\begin{array}{c} X_{1}^{\text{T}},t^{\left(b\right)},\alpha^{\left(b\right)}\\ \vdots \\ X_{N_{\text{nodes}}}^{\text{T}},t^{\left(b\right)},\alpha^{\left(b\right)} \end{array}\right].$$

Each row corresponds to a single nodal query, while the loading step and loading ratio remain fixed across all the nodes for that batch element. The neural network operated point-wise on the rows of $Z^{\left(b\right)}$ and output a matrix of predictions for the nodal displacements. Thus,

(18) $${\hat{u}}^{\left(b\right)}=N_{\theta }\left(Z^{\left(b\right)}\right)\in R^{N_{\text{nodes}}\times 2},$$

where the $i^{\text{th}}$ row ${\hat{u}}_{i}^{\left(b\right)}={\left(U_{1,i},U_{2,i}\right)}^{T}$ denotes the NN-FE predicted displacement components at node i. Overall, this result defines a complete nodal displacement field associated with the sampled loading state $\left(t^{\left(b\right)},\alpha^{\left(b\right)}\right)$, which is generally statically admissible. Using the standard FE interpolation, the predicted nodal displacements define a continuous displacement field ${\hat{u}}^{\left(b\right)}(X)$. Then, Abaqus evaluates the constitutive response through the first PK stress P(F), assembles the residual forces, and solves the resulting nonlinear equilibrium, i.e. Eq. (16), under the total Lagrangian formulation together with Newton-Raphson iterations, yielding a statically admissible displacement field at the subsequent increment as

(19) $$u^{*(b)}\left(t^{\left(b\right)}+\Delta t,\alpha^{\left(b\right)}\right)=A\left({\hat{u}}^{\left(b\right)},\Delta \underline{u}\right).$$

Herein, A(⋅) is the nonlinear FE equilibrium operator and $\Delta \underline{u}$ represents the experimentally prescribed incremental displacement boundary conditions. Within this NN–FE framework, the operator A(⋅) was treated as a non-differentiable black box, and no gradients were propagated through the equilibrium solver. End-to-end differentiation through the solver would require explicit access to its computational graph, thereby restricting the approach to differentiable, Python-based implementations. While inherently differentiable FE suites (e.g., JAX-FEM, FEniCS) allow analytical propagation of sensitivity gradients from the equilibrium equations directly back to the network parameters θ, they require this explicit computational graph access. By deliberately avoiding this requirement, the proposed NN–FE framework remains independent of the numerical implementation of the FE solver and can directly integrate both commercial and open-source FE codes (e.g., FEBio [45]) without significant modifications.

Next, the FE-corrected displacement $u^{*(b)}$ served as a physics-consistent reference for the corresponding batch element. The neural network was then evaluated again at the same nodal coordinates for the updated loading step, and agreement between ${\hat{u}}^{\left(b\right)}$ and $u^{*(b)}$ was enforced via a physics-based loss function. The total loss for the iteration was obtained by averaging over all batch elements, which enables the neural network to learn displacement fields that are consistent with the equilibrium behavior of the underlying hyperelastic material, while simultaneously maintaining computational efficiency.

### 3.4 Physics-based loss function and training procedure

The training objective function ${ℒ}_{\text{phys}}$ was defined by the mean squared difference between the neural network predictions and the FE-corrected displacement fields over all batch elements as

(20) $${ℒ}_{\text{phys}}=\frac{1}{N_{\text{batch}}}\sum_{b=1}^{N_{\text{batch}}}\|{\hat{u}}^{\left(b\right)}\left(X,t^{\left(b\right)}+\Delta t,\alpha^{\left(b\right)}\right)-u^{{*}^{\left(b\right)}}(X,t^{\left(b\right)}+\Delta t,\alpha^{\left(b\right)}){\|}_{2}.$$

Minimization of the training objective function ${ℒ}_{\text{phys}}$ with respect to the neural network parameters θ was performed using the standard stochastic gradient optimization, enabling the neural network to learn the displacement fields that are consistent with the underlying quasi-static hyperelastic behavior, while maintaining computational efficiency.

## 4. Experimental Data Acquisition and Simulation Data Generation

### 4.1 Experimental data

#### 4.1.1 Tissue preparation

**Figure 4 –:**
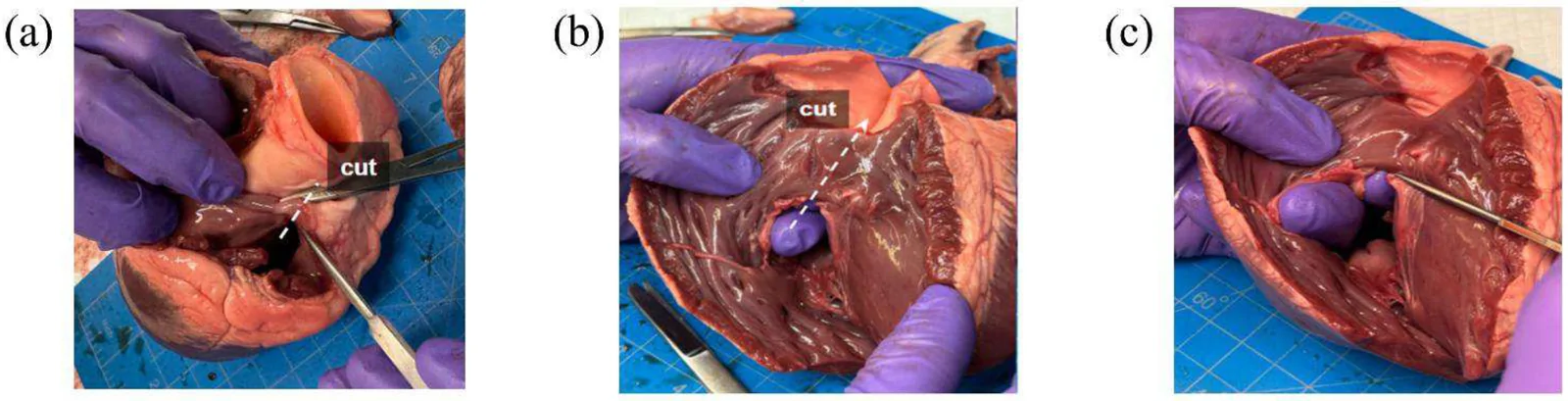
Sequential dissection protocol for isolation of the porcine tricuspid valve posterior leaflet: (a) initial incision through the right atrium between tricuspid valve leaflets to expose the valve apparatus; (b) superior view showing the separation cut between anterior and posterior leaflets; (c) upward incision to complete leaflet separation.

The tricuspid valve and the posterior leaflet were obtained from the heart of an adult pig (age 1.5 weight 120 kg), sourced from a USDA-approved supplier (Animal Technologies, TX, USA). Upon arrival at the lab, the heart was cleaned of residual blood and immediately stored at −20 °C. For dissection and valve leaflet excision, the heart was thawed at room temperature. Following our prior protocol, dissection began by removing excess pericardium and cutting the lower third of the ventricles to distinguish the right and left sides of the heart based on wall thickness [46]. The atria were then excised to expose the tricuspid and mitral valves, and a careful incision between the leaflets using a razor blade fully opened the right and left ventricles. From this dissection, the TVPL was isolated (Fig. 4), preserved in phosphate-buffered saline (PBS), and temporarily stored at −20 °C until testing. This short-term frozen storage approach is supported by previous findings, which demonstrated that brief freezing has minimal impact on the measured mechanical properties of collagenous tissue [47,48].

For biaxial mechanical testing, the TVPL tissue was thawed by submerging in deionized water at room temperature, and a 10 × 10 mm specimen was excised from the central, belly region. Tissue thickness was measured at three locations using a laser thickness gauge (Keyence IL-030, Itaska, IL, USA); the mean value was 0.28 mm. Subsequently, the specimen was mounted in the BioTester 5000 system (CellScale, Canada), with each edge secured using 5-tine BioRakes. Finally, the specimen was submerged in a 37 °C PBS bath to simulate the physiological conditions of the valve for the subsequent biaxial mechanical testing [37].

#### 4.1.2 Biaxial testing protocol

For biaxial testing, experiments were conducted according to a protocol that—with some modifications—resembled that of earlier studies [37, 49]. In brief, the peak force $F^{\text{peak}}$ for the TVPL specimen was calculated from the target first PK stress $P^{\text{target}}$, the initial effective edge length $L_{0}$, and the undeformed thickness $t_{0}$, using the formula $F^{\text{peak}}=P^{\text{target}}\times L_{0}\times t_{0}$. The target stress values $P^{\text{target}}$ of 125, 250 and 500 kPa were chosen based on the load cell’s maximum capacity of 1.5 N, with a safety limit of 1.4 N. We began with a target stress of 500 kPa and, if the corresponding peak force $F^{\text{peak}}$ in either the X- or Y-direction exceeded this force safety limit, the target stress was halved iteratively until the resulting force fell within the safety threshold. In this experiment, the target stress of 500 kPa was achieved for the specimen, resulting in peak forces $F_{X}^{\text{peak}}$ and $F_{Y}^{\text{peak}}$ of 760.8 mN.

After determining the target peak forces, the TVPL specimen was then preconditioned to approximate its *in vivo* pretension. The preconditioning protocol consisted of eight force-controlled loading/unloading cycles, each bringing the specimen to its target force in the X- and Y-directions over 30 s, and then returning it to its initial state over the following 30 s. The loading rate was chosen to promote uniform mechanical behavior and reduce the hysteresis effect (i.e., quasi-static testing conditions) [37]. Following preconditioning, the $\Omega_{\text{PPC}}$ configuration was recorded for the subsequent analysis of tissue stretch and mechanical stress.

Next, the tissue was subjected to seven varying biaxial loading ratios $\left(F_{X}:F_{Y}=1:1,1:0.75,1:0.5,1:0.25,0.75:1,0.5:1,0.25:1\right)$, with each test starting from the post-preconditioning configuration $\Omega_{\text{PPC}}$. These tests were carried out to determine the biaxial displacements and the time required for the tissue to reach, in each direction, its respective force level—either the full target force or 75%, 50%, or 25% of that value—according to the specified loading ratio [37,49]. The resulting displacement–time profiles were then used to define the loading sets for the actual displacement-controlled tests. After defining the displacement-controlled sets, the tissue underwent biaxial mechanical characterization, consisting of six stretch-recovery cycles under each biaxial loading ratio, performed at maximum velocity and acceleration limits of 2 mm/s and 2 mm/s^2^, respectively, to approximate quasi-static loading conditions. A rest period of 120 s was used between any two loading ratios to allow the tissue to return to its post-preconditioning state and to ensure consistent initial conditions for all ratios.

Data from these loading cycles were used in stress/strain analysis, constitutive modeling and parameter estimation, and Abaqus FE simulations.

### 4.2 Simulated data

#### 4.2.1 Estimation of the Fung model parameters

The goal of parameter identification is to determine the set of model parameters $\theta =\{c,b,\kappa {\}}^{\text{T}}$ that best reproduce the experimentally measured in-plane nominal stresses ${\left\{P_{11}^{\text{exp},(i)},P_{22}^{\text{exp},(i)}\right\}}_{i=1}^{N}$ across all experimental data points. For each experimental set of data, the out-of-plane stretch $\lambda_{3}^{\left(i\right)}$ is analytically determined by enforcing the plane-stress condition according to

(21) $$S_{33}\left(\lambda_{1}^{\left(i\right)},\lambda_{2}^{\left(i\right)},\lambda_{3}^{\left(i\right)};\theta \right)=0,$$

which leads to the solution of the out-of-plane stretch as

(22) $$\lambda_{3}^{\left(i\right)}=f\left(\lambda_{1}^{\left(i\right)},\lambda_{2}^{\left(i\right)};\theta \right).$$

The in-plane nominal stresses are then computed using the corresponding principal stretches. Thus,

(23) $$P_{11}^{\text{model,}(i)}(\theta )=\lambda_{1}^{\left(i\right)}S_{11}\left(\lambda_{1}^{\left(i\right)},\lambda_{2}^{\left(i\right)},\lambda_{3}^{\left(i\right)};\theta \right),\;P_{22}^{\text{model,}(i)}(\theta )=\lambda_{2}^{\left(i\right)}S_{22}\left(\lambda_{1}^{\left(i\right)},\lambda_{2}^{\left(i\right)},\lambda_{3}^{\left(i\right)};\theta \right),$$

where $S_{11}$ and $S_{22}$ are the normal components of the second PK stress, defined in Sections 2.1 and 2.3.

The optimal parameters $\overset{ˆ}{\theta }$ minimize the sum of squared errors between the stresses predicted by the model and the experimental measurements, i.e.

(24) $$\overset{ˆ}{\theta }=arg\;\underset{\theta }{\text{min}}\;R(\theta ),\;R(\theta )=\sum_{i=1}^{N}\left[{\left(P_{11}^{\text{model,}(i)}(\theta )-P_{11}^{\text{exp},(i)}\right)}^{2}+{\left(P_{22}^{\text{model,}(i)}(\theta )-P_{22}^{\text{exp},(i)}\right)}^{2}\right],$$

where R(θ) represents the objective function representing the sum of squared errors between the model-predicted and experimentally measured stress components, and $\overset{ˆ}{\theta }$ denotes the optimal parameter set that minimizes R(θ). Due to the exponential form of the Fung’s strain-energy function and the implicit plane-stress constraint, this optimization problem is highly nonlinear. To solve this nonlinear optimization problem, a custom differential evolution optimization algorithm [50] was implemented in MATLAB with Weibull-flight mutations [51] to enhance global exploration. The algorithm optimizes the eight model parameters in base-10 logarithmic space: the coefficient c, the bulk modulus κ, and six independent entries of the symmetric 6 × 6 matrix b (expressed in Voigt’s notation). Initial parameter values were randomly selected in log-space within bounds corresponding to the range [0.001, 500] on a linear scale; a population size of 40, a crossover rate of 0.75, and a maximum of 100 000 function evaluations were used, with early termination occurring if R(θ)<0.3. The algorithm employed a staged strategy that transitions from global exploration to local exploitation: i.e., early-stage Weibull-based exploration (evaluations≤ 30%), middle-stage adaptive mixing (30–70%), and final-stage refinement around the best solution (evaluations> 80%). Out-of-bound parameters were bounded using midpoint constraints. Each candidate solution was validated for positive definiteness via eigenvalue decomposition of the full 6 × 6 matrix b; solutions with any eigenvalue below 1 × 10^−6^ were rejected by assigning an infinite cost. For admissible parameters, R(θ) was evaluated by solving $S_{33}=0$ for $\lambda_{3}^{\left(i\right)}$ at each data point using MATLAB’s fzero function, then computing the predicted stresses via Eqs. (10)–(13).

**Figure 5 –:**
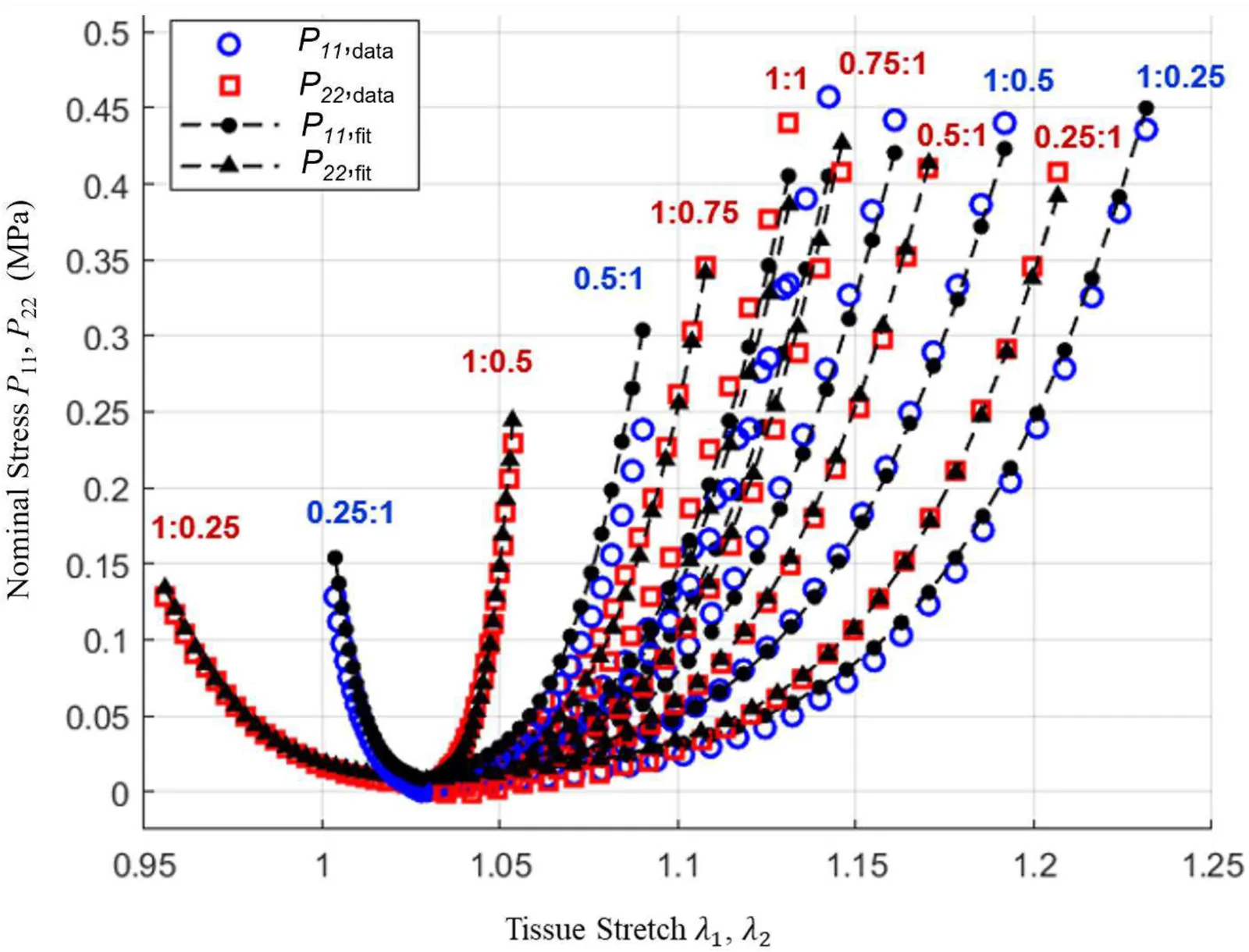
Experimental validation of Fung’s hyperelastic model across all seven loading ratios. Open circles $\left(P_{11,\text{data}}\right)$ and black dashed curves with circles $\left(P_{11,\text{fit}}\right)$ show the circumferential direction; open squares $\left(P_{22,\text{data}}\right)$ and black dashed curves with triangles $\left(P_{22,\text{fit}}\right)$ show the radial direction.

All seven experimental loading ratios were considered for determining the parameters of the Fung model; however, only the unloading portions of the final two cycles of each ratio were used for calibration. This choice accounts for the fact that the initial cycles and loading segments are influenced by preconditioning, viscoelastic effects, stress softening, and microstructural rearrangements, whereas the unloading during the final cycles exhibits stabilized, path-independent elastic response consistent with the assumptions of the Fung model [52]. The corresponding parameters are listed in Table 1, and the model fits for all loading ratios are shown in Fig. 5.

#### 4.2.2 Abaqus finite element model

A FE model of the specimen was developed in Abaqus (Dassault Systèmes, RI, USA) to simulate the mechanical response for each $F_{X}:F_{Y}$ loading ratio. Given the high aspect ratio of the valvular tissue specimen (thickness: ~0.2 mm vs planar dimension: 10 mm), the tissue was modeled as a planar structure using four-node, quadrilateral shell elements with reduced integration (S4R). A mesh convergence study was performed (see Appendix A) to determine the optimal element density.

To model pure planar deformation, the out-of-plane displacement $U_{3}$ and rotations about the global X and Y axes, i.e. $UR_{1},UR_{2}$, were constrained for all nodes. To eliminate rigid-body motions, a single node at the specimen center was pinned in both the X- and Y-directions. The experimental BioRakes interface was modeled using five reference points (RPs) per edge, corresponding to the physical tines. Each RP was linked to the mesh via a kinematic coupling to a four-node element; the choice of kinematic coupling was motivated by a comparative study between kinematic and distributing couplings presented in Appendix B. The boundary conditions were prescribed using the experimentally measured displacement-time profiles, implemented via tabulated amplitudes to replicate the experimental biaxial loading history.

For the FE simulation and the subsequent training of the neural network, only the duration of a single complete cycle per loading ratio was considered. This choice is based on the assumption that no energy dissipation occurs in the FE model—resulting in identical, repeating cycles (i.e., no viscous effects) [53]—so that it is not necessary to train the neural network model using multiple identical cycles.

### 4.3 Case study design and neural network training strategy

To evaluate the generalization capability of the proposed hybrid NN-FE framework, a leave-one-ratio-out cross-validation strategy was employed across six different case studies. The naming convention reflects the loading ratio *excluded* from training and is supplemented by a classification into in-distribution and out-of-distribution categories based on the training data composition.

For the four in-distribution (ID) case studies, the model was trained on six out of the seven available loading ratios, with one asymmetric ratio held out for testing. For example, in-distribution Case Study ID-1:0.75 was trained on the loading ratios $F_{X}:F_{Y}=1:1,1:0.5,1:0.25,0.75:1,0.5:1,\;\text{and}\;0.25:1$, while the $F_{X}:F_{Y}=1:0.75$ loading ratio was reserved for testing. Similarly, Case Study ID-1:0.5 excluded the $F_{X}:F_{Y}=1:0.5$ ratio from training, Case Study ID-0.75:1 excluded the $F_{X}:F_{Y}=0.75:1$ ratio, and Case Study ID-0.5:1 excluded the $F_{X}:F_{Y}=0.5:1$ ratio. In each in-distribution case, the equibiaxial 1:1 ratio was always included in the training set. These in-distribution cases assess the ability of our NN-FE framework to interpolate mechanical behavior for asymmetric loading conditions similar to those encountered during training.

On the other hand, for the two out-of-distribution (OOD) case studies the equibiaxial loading ratio $\left(F_{X}:F_{Y}=1:1\right)$ was held out for testing, providing a more rigorous evaluation of the model’s extrapolation capability. To ensure consistency and assess directional coupling due to material anisotropy, two distinct training configurations were considered: Case Study OOD-1:1(A) trained exclusively on X-dominant ratios $\left(F_{X}:F_{Y}=1:0.75,1:0.5,1:0.25\right)$, while Case Study OOD-1:1(B) trained exclusively on Y-dominant ratios $\left(F_{X}:F_{Y}=0.75:1,0.5:1,0.25:1\right)$. This asymmetric training strategy evaluates whether the NN-FE framework can accurately extrapolate to equibiaxial loading when trained only on data exhibiting preferential loading in one direction. The distinct training sets for the two OOD cases also enable assessment of potential directional dependencies/coupling in the learned stretch-stress relationships.

Each case was trained independently using the solver-in-the-loop approach described in Section 3.3, with training convergence and performance evaluated based on the held-out loading ratio to quantify generalization accuracy.

## 5. Results

The tissue specimen is modeled as a square planar domain of total size 10 × 10 mm, with in-plane dimensions X=7.54mm and =7.26mm, corresponding to the distances between opposing right–left and top–bottom BioRakes tines in the experiment and between RPs in the model. The tissue was treated as an anisotropic, nearly incompressible hyperelastic solid and simulated using a 3D shell planar FE model in ABAQUS. Given the thin geometry of the TVPL (t=0.277mm), the specimen was discretized by four-node quadrilateral shell elements with reduced integration (S4R), whereby near-incompressibility is enforced by the choice of Poisson’s ratio v=0.499. Quasi-static deformation was applied through displacement-controlled boundary conditions derived directly from experimentally measured biaxial displacement–time profiles. Seven biaxial loading ratios were considered, corresponding to varying force proportions along the circumferential and radial directions. Each loading case was applied incrementally over a full loading cycle. The resulting FE-predicted displacement and stress fields were used within a hybrid FE–NN framework to train the model and predict tissue stress response under biaxial loading. The choices of FE modeling, including mesh refinement, RP connections to tissue, and contact coupling definitions, were determined through a separate parametric study (see Appendices A and B). The neural network architecture and associated hyperparameters were selected based on a systematic evaluation of tissue network configurations, including the number of hidden layers, neurons per layer, and choice of activation function.

### 5.1 Training behavior and convergence

**Figure 6 –:**
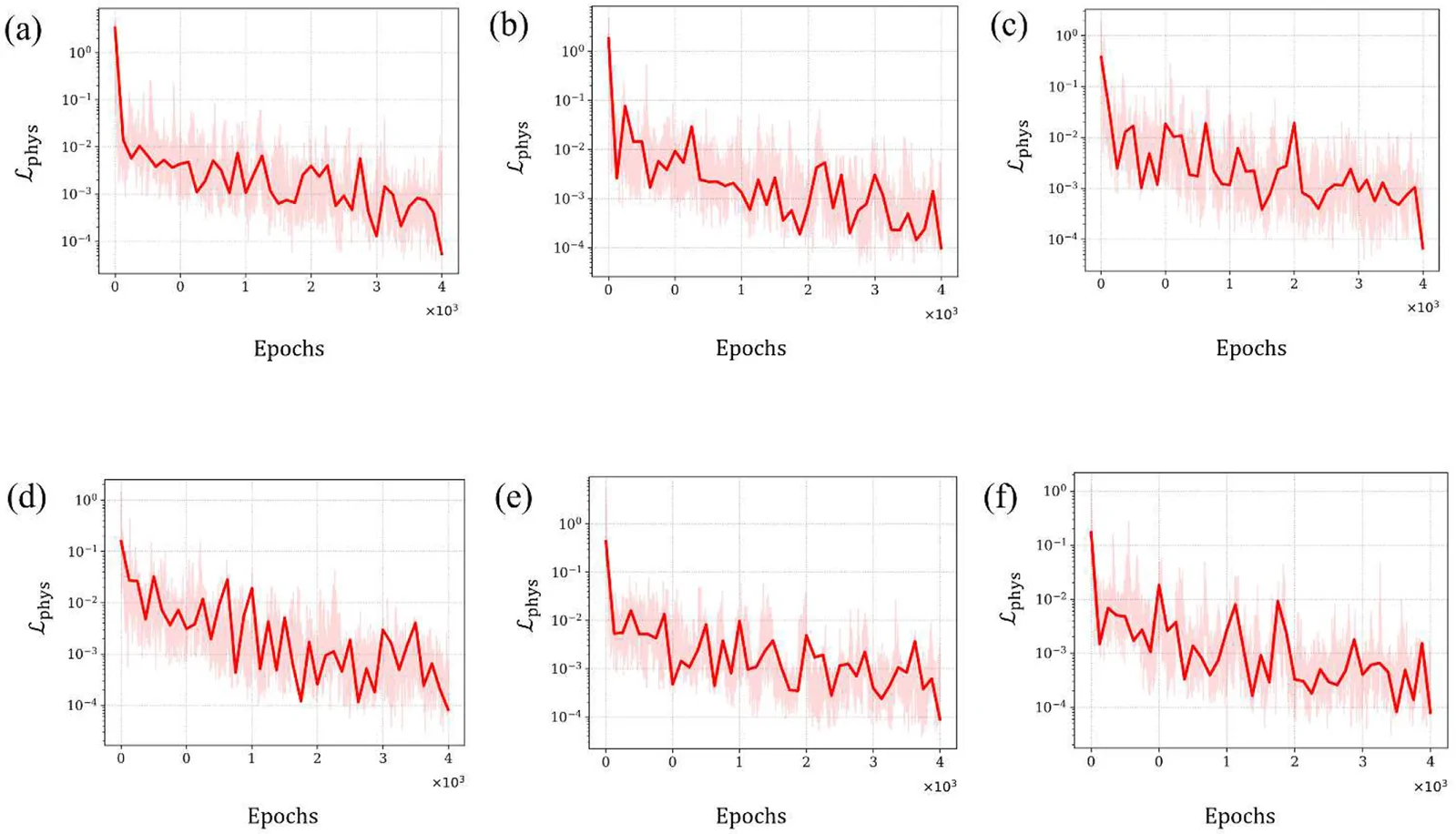
Training loss histories for the six case studies: (a) ID-1:0.75, (b) ID-1:0.5, (c) ID-0.75:1, (d) ID-0.5:1, (e) OOD-1:1(A), and (f) OOD-1:1(B). Thin colored curves represent the instantaneous total loss values per epoch, and thick red curves denote 100-epoch moving average. ID = in-distribution; OOD = out-of-distribution.

The convergence behavior of the proposed hybrid FE–NN framework during training is shown in Fig. 6 for all six studies case studies, applying the leave-one-loading-ratio-out principle. For each case, the reported loss value corresponds to the physics motivated discrepancy between the displacement fields predicted by the neural network and the FE-corrected displacement fields, evaluated based on the training dataset. Across all case studies, the training loss decreases consistently and converges to a stable plateau without divergence. While the instantaneous loss exhibits oscillations due to mini-batch sampling and the solver-in-the-loop data generation, the moving-average loss (computed over 100 epochs) demonstrates clear monotonic convergence. For all cases, the averaged loss stabilizes within approximately $𝒪\left(10^{-4}\right)$ after ~2 × 10^4^ training epochs, with final values remaining within the same order of magnitude across different held-out loading ratios. The consistent convergence observed for all training configurations indicates that the proposed solver-integrated training strategy provides robust optimization stability for strongly nonlinear hyperelastic response, despite the absence of direct gradient propagation through the FE equilibrium solver.

### 5.2 Spatial displacement fields at peak loading

**Figure 7 –:**
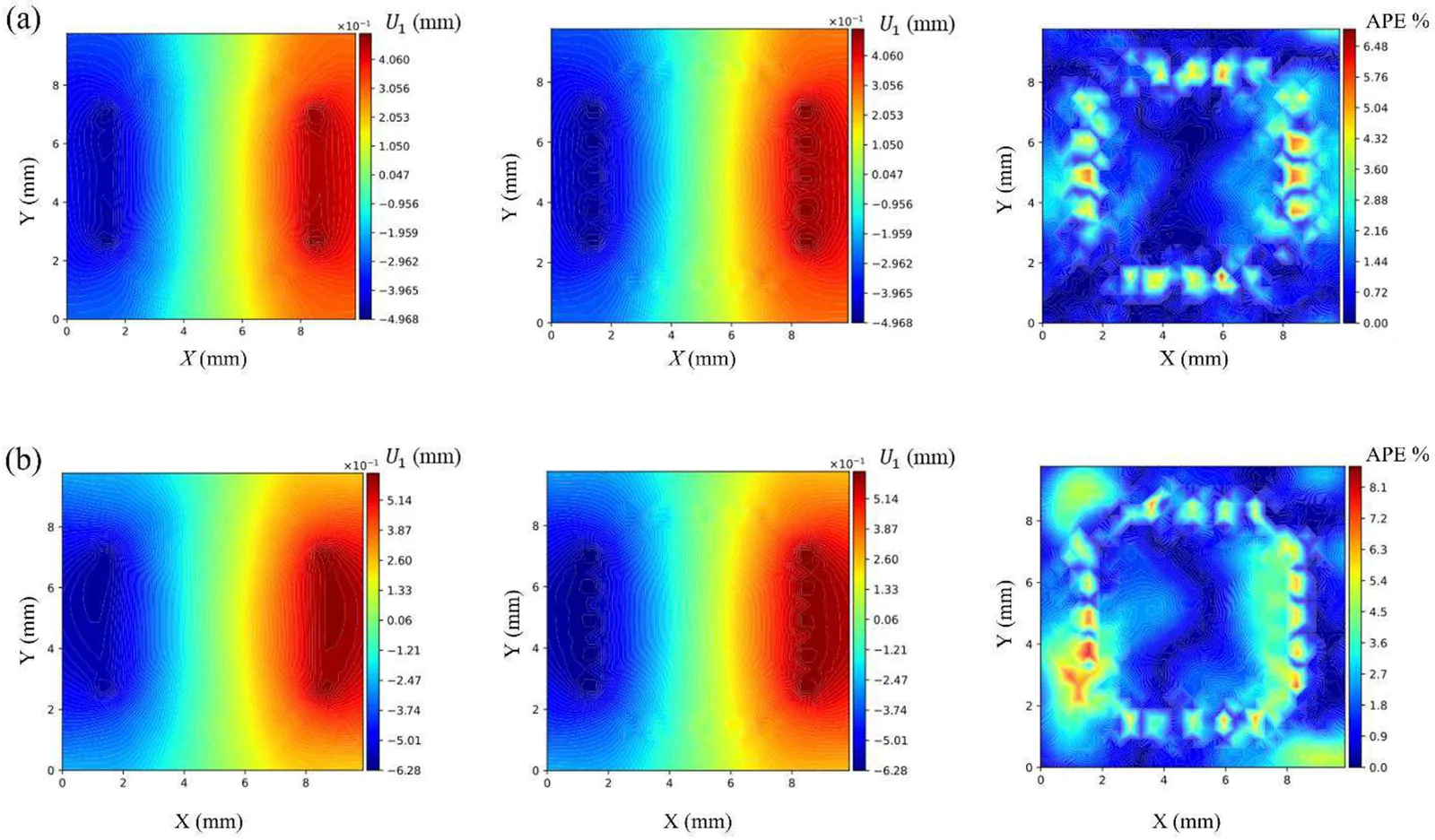

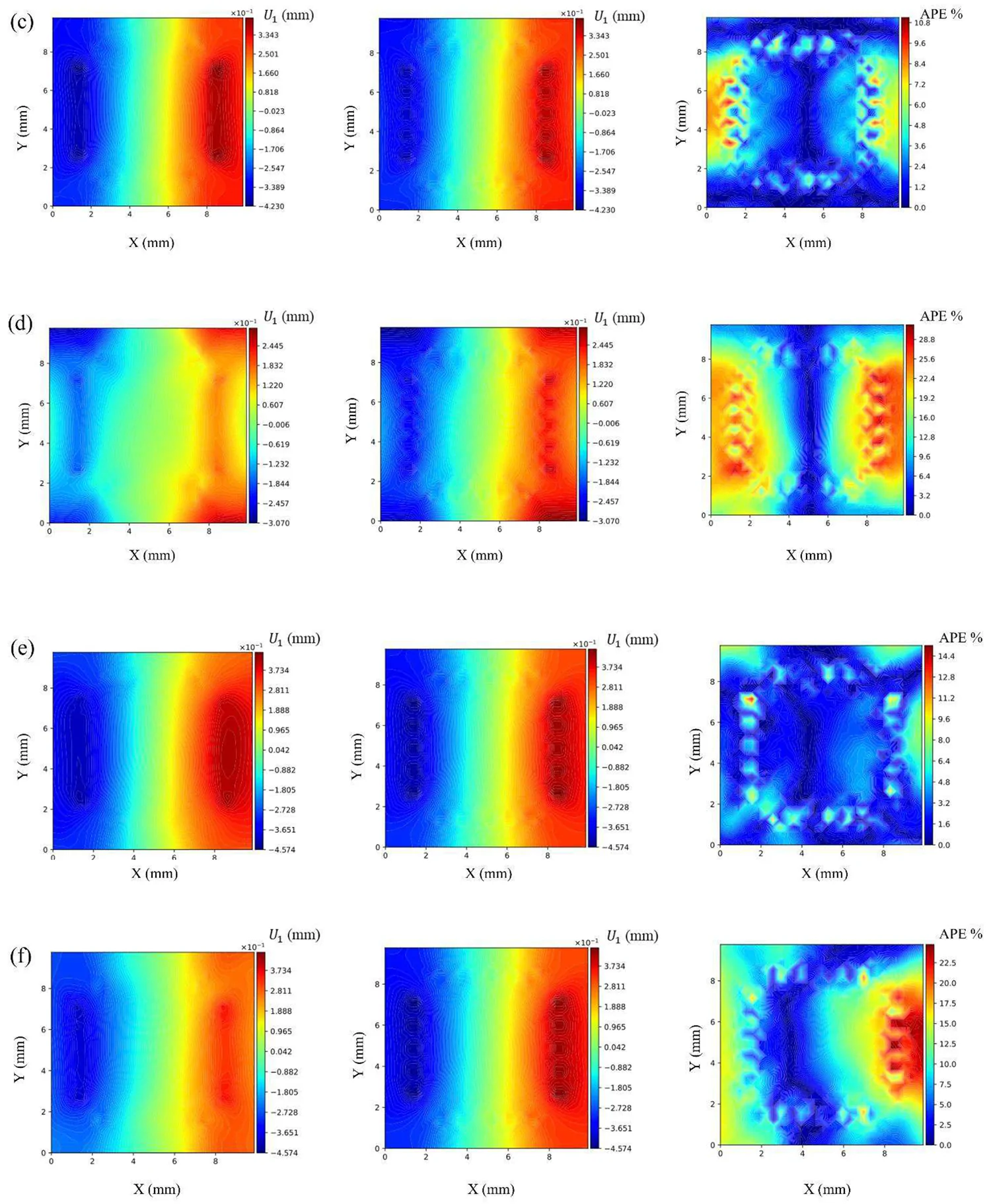
Comparison of X-displacement $U_{1}$ contours at the peak loading. Columns 1 to 3 represent the NN–FE model prediction, FE solution, and absolute relative percentage error, respectively. Rows correspond to the distinct held-out loading ratios: (a) ID-1:0.75, (b) ID-1:0.5, (c) ID-0.5:1, (d) ID-0.75:1, (e) OOD-1:1(A), and (f) OOD-1:1(B).

**Figure 8 –:**
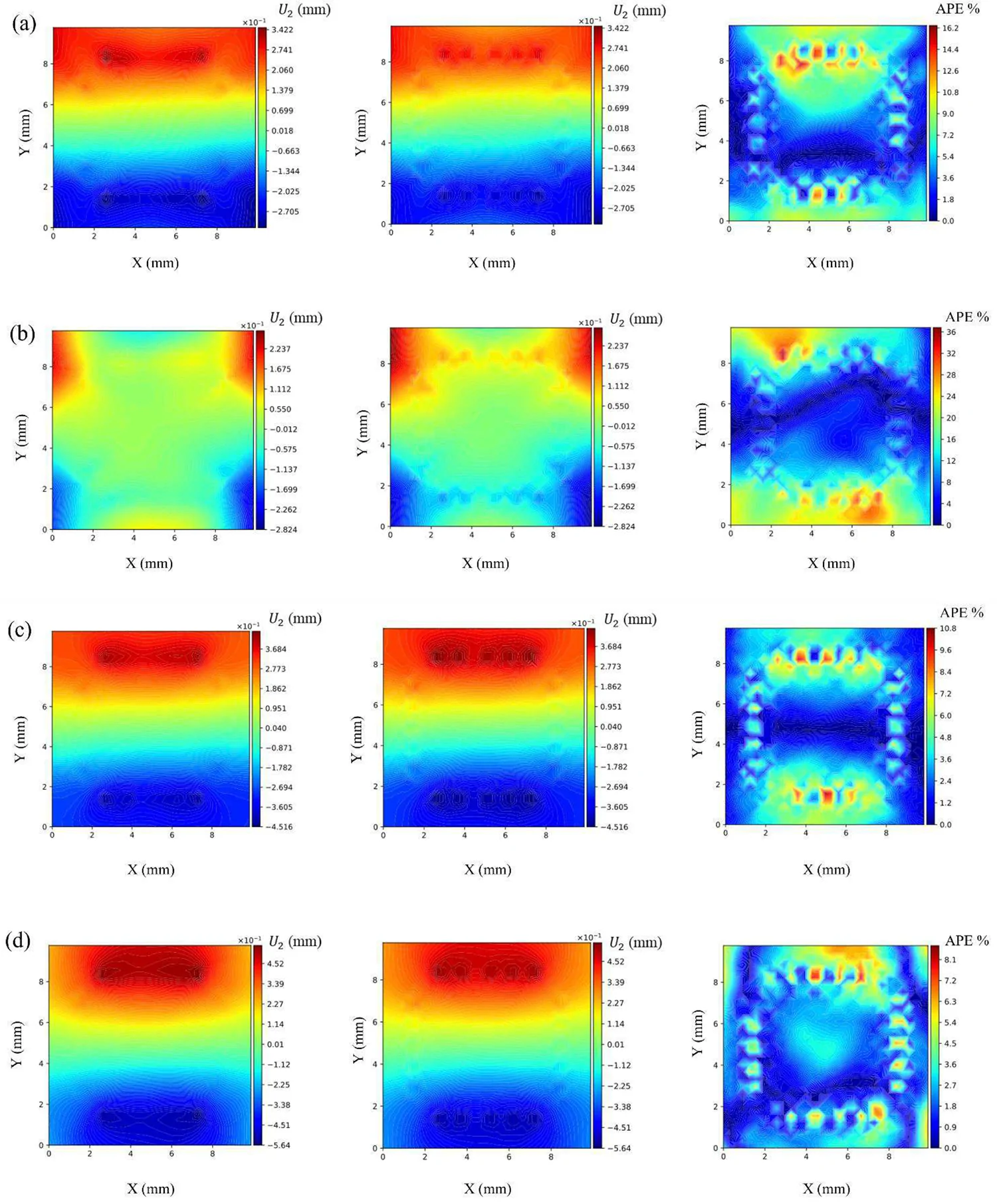

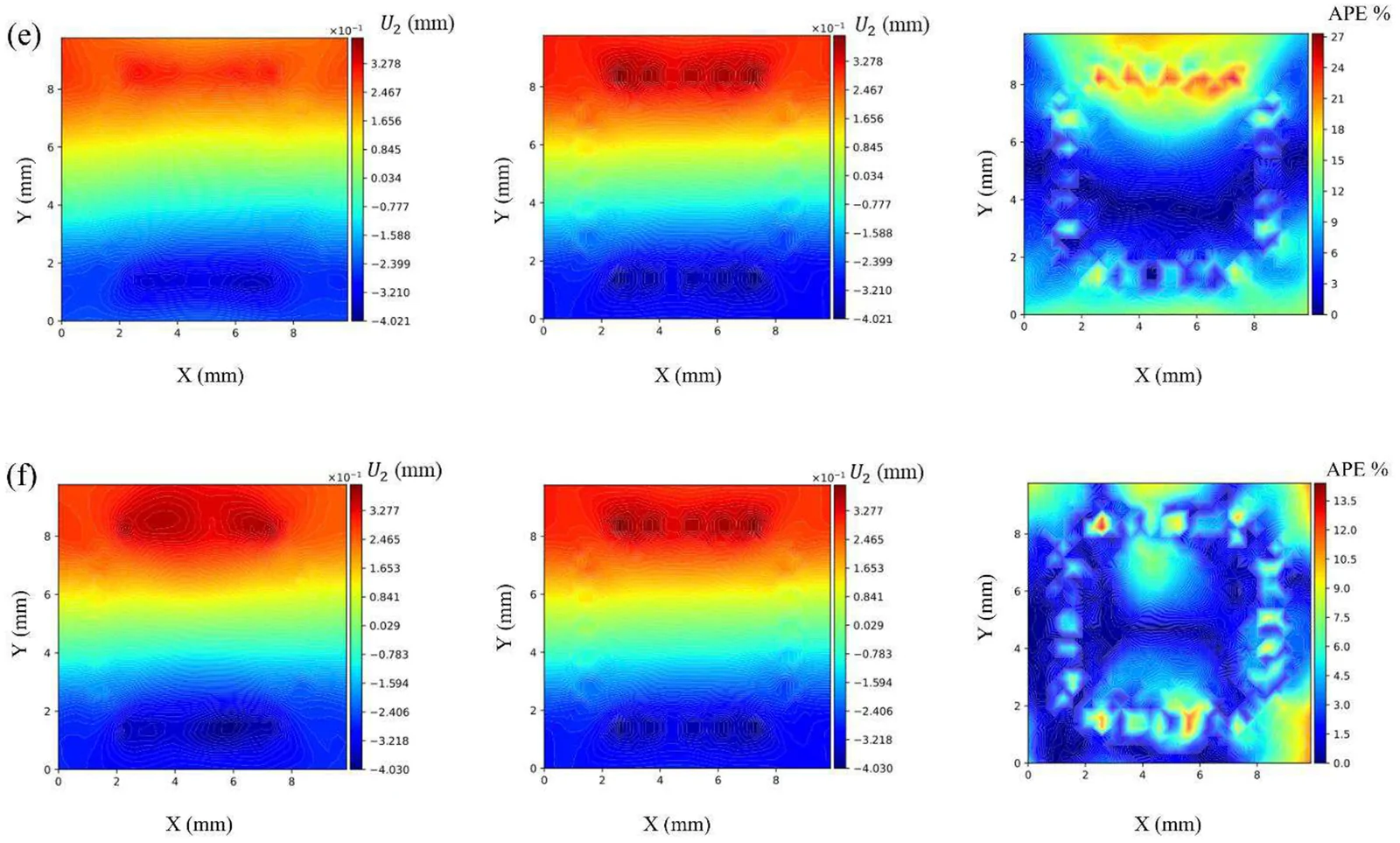
Comparison of Y-displacement $U_{2}$ contours at the peak loading. Columns 1 to 3 represent the NN–FE model prediction, FE solution, and absolute relative percentage error, respectively. Rows correspond to the distinct held-out loading ratios: (a) ID-1:0.75, (b) ID-1:0.5, (c) ID-0.5:1, (d) ID-0.75:1, (e) OOD-1:1(A), and (f) OOD-1:1(B).

Spatial comparisons of predicted and ground-truth displacement fields ($U_{1}$ and $U_{2}$) at peak loading are shown in Figs. 7 and 8, respectively. Each row corresponds to a distinct case study in which the tested loading ratio was completely excluded from the training dataset in order to evaluate the framework’s generalization capability under previously unseen biaxial loading conditions. The first column displays the NN–FE predicted displacement contours, the second column shows the FE reference solution, and the third column presents the pointwise absolute percentage error (APE) distribution (for a definition, see Appendix C).

For the $U_{1}$ displacement component (Fig. 7(a)–(f)), the framework achieves root mean square errors (RMSE) (for a definition, see Appendix C) ranging from 0.0083 mm to 0.0517 mm across all case studies (Table 2). The mean absolute percentage error (MAPE) (for a definition, see Appendix C) varies from 1.24% for Case Study ID-1:0.75 to 14.9% for Case Study ID-0.5:1. Maximum localized errors range from 6.81% (ID-1:0.75) to 31.0% (ID-0.5:1), with the OOD case study OOD-1:1(A) exhibiting a maximum error of 24.5%. The error distribution contours reveal that predictions maintain high accuracy throughout the interior tissue domain, with elevated errors concentrated near the constrained boundaries and BioRakes loading interfaces.

For the $U_{2}$ displacement component (Fig. 8(a)–(f)), RMSE values span 0.0154 mm to 0.0408 mm (Table 3). The MAPE ranges from 2.44% (ID-0.5:1) to 12.2% (ID-1:0.5), with maximum localized errors between 8.62% (ID-0.5:1) and 36.6% (ID-1:0.5). The OOD case studies exhibit maximum errors of 14.4% for OOD-1:1(A) and 27.1% for OOD-1:1(B). Similar to the $U_{1}$ component, the error distribution contours demonstrate that the largest discrepancies are localized at the specimen edges, while the bulk tissue region shows substantially lower percentage errors, typically under 10%.

### 5.3 Time-dependent displacement response

While the contour plots in Section 5.2 provide spatial snapshots of the proposed hybrid NN–FE model’s performance at peak load, the temporal evolution of the displacement fields throughout the entire loading cycle is equally critical for validating the solver-in-the-loop framework. To evaluate global tracking accuracy, the root mean square (RMS) of nodal displacements was computed for the $U_{1}$ and $U_{2}$ components.

**Figure 9 –:**
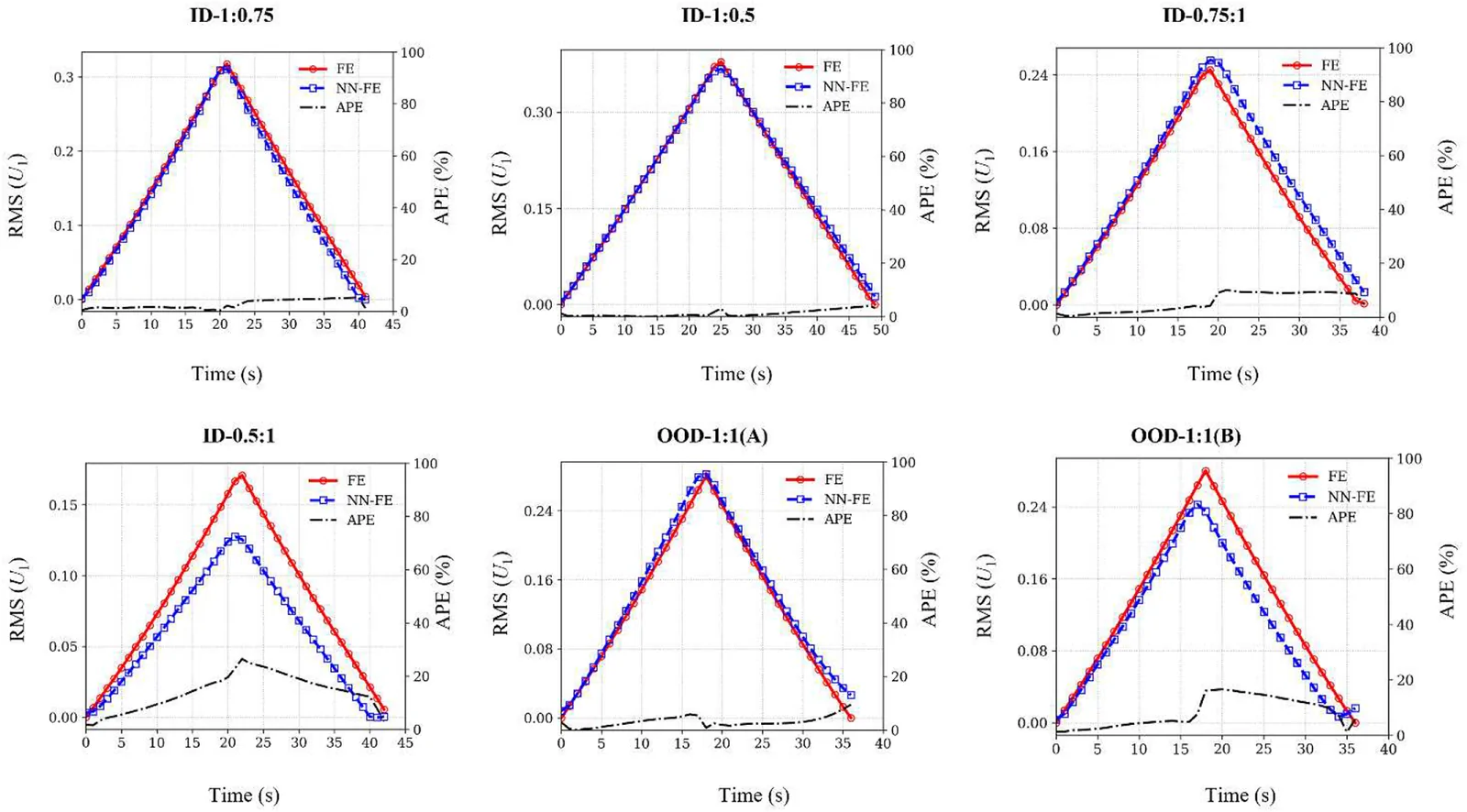
X-displacement response $U_{1}$ for different case studies, in terms of the time-dependent root mean square (RMS) value.

**Figure 10 –:**
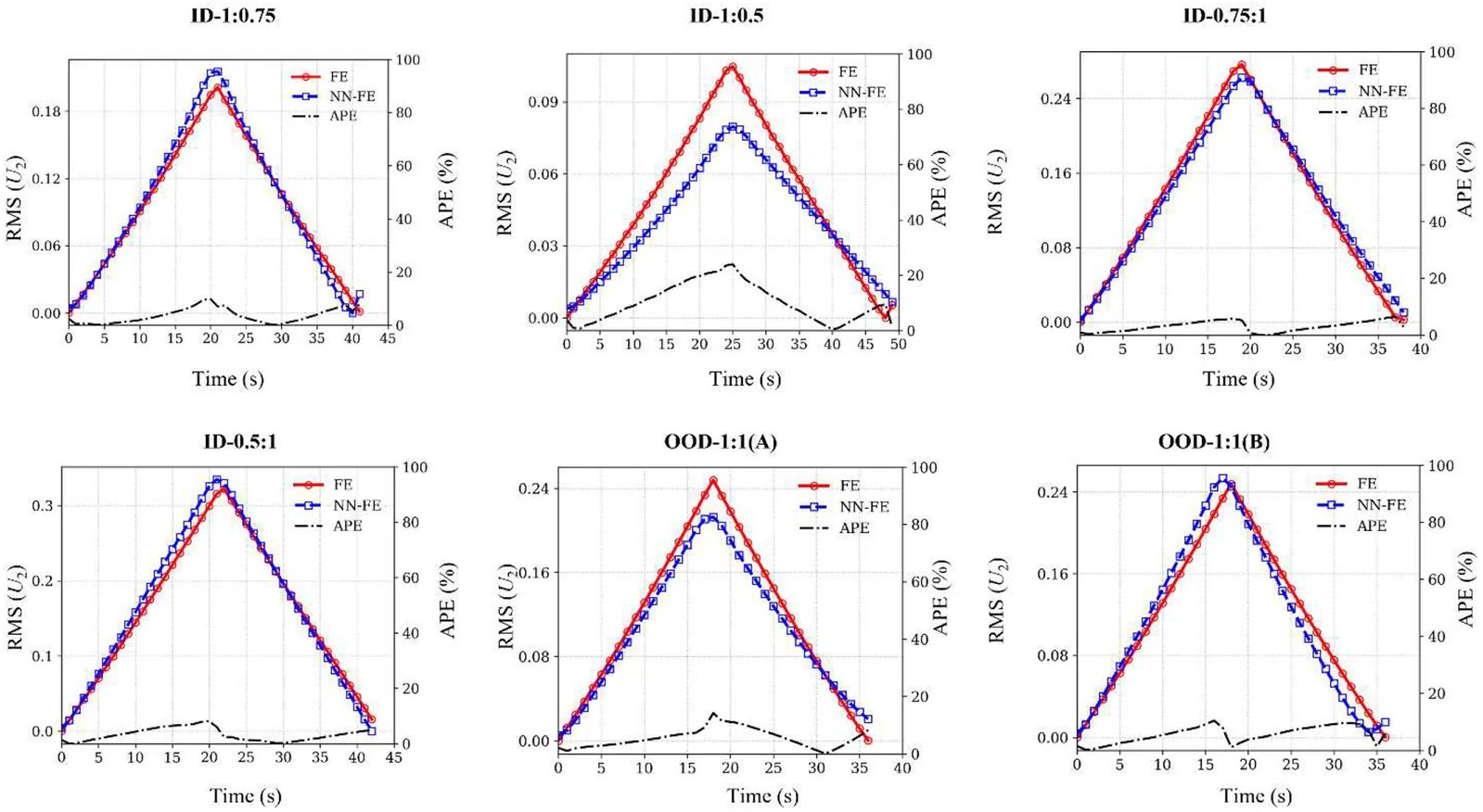
Y-displacement response $U_{2}$ for different case studies, in terms of the time-dependent root mean square (RMS) value.

The comparisons between the RMS displacement predicted by the NN–FE framework (blue curves) and the ground-truth FEM solutions (red curves) across the full loading and unloading sequence for the $U_{1}$ and $U_{2}$ components are summarized in Figs. 9 and 10, respectively. The black dashed curves indicate the APE, referenced to the positive Y-axis. Quantitative assessment of the temporal tracking accuracy is provided through the coefficient of determination $R^{2}$ (for a definition, see Appendix C) and the maximum APE values in Table 4.

For the $U_{1}$ component (Figs. 9(a)–(f)), the framework demonstrates strong temporal synchronicity across most case studies, with $R^{2}$ values ranging from 0.7248 (ID-0.5:1) to 0.9969 (ID-1:0.5). The in-distribution cases ID-1:0.75 and ID-1:0.5 exhibit particularly strong agreement with $R^{2}$ values of 0.9884 and 0.9969, respectively. The out-of-distribution cases OOD-1:1(A) and OOD-1:1(B) achieved $R^{2}=0.8922$ and $R^{2}=0.9541$, respectively, demonstrating robust generalization to the equibiaxial loading condition. Across all cases, the maximum APE for $U_{1}$ ranges from 3.92% (ID-1:0.5) to 26.5% (ID-0.5:1) (Table 4), with the peak errors consistently occurring near the maximum load range.

For the $U_{2}$ component (Fig. 10(a)–(f)), temporal tracking accuracy remains strong, with R^2^ values spanning 0.8351 (ID-1:0.5) to 0.9868 (ID-0.75:1). The ID-1:0.75, ID-0.75:1, ID-0.5:1, and OOD-1:1(A) cases show particularly high fidelity, with R^2^ values exceeding 0.95. Maximum APE for U_2_ ranges from 6.41% (ID-0.75:1) to 24.0% (ID-1:0.5), as detailed in Table 4.

In several instances—particularly in the OOD scenarios and Case Study ID-0.5:1—a slight temporal phase shift is observed. This phenomenon, reflected in the somewhat lower $R^{2}$ values for these cases, represents a known challenge when training neural networks for time-dependent physical processes; it typically stems from the model’s difficulty in adequately capturing high-frequency transitions or rapid changes in the loading rate. Despite these phase deviations, maximum temporal errors remain below 27% in all cases; furthermore, trajectory analysis confirms that the solver-in-the-loop architecture successfully regularizes the network’s predictions, thereby maintaining physical consistency across the dynamic loading profile.

### 5.4 Time-dependent stress response

Beyond kinematic tracking, the framework’s ability to predict the temporal evolution of internal stress states provides a critical validation of its physical consistency. The neural network does not directly output stress values; instead, nodal displacement predictions are used to derive the corresponding element-wise Cauchy stress components $\sigma_{11}$ and $\sigma_{22}$ through the Fung-type constitutive relationship. This post-processing step serves as a rigorous physical validation, ensuring that the network’s predicted displacements yield stress fields consistent with the ground-truth FE solutions. To provide a global comparison, the volume-averaged Cauchy stress for each component was calculated as

(25) $${\underline{\sigma }}_{kk}=\frac{\sum_{j=1}^{M}\sigma_{kk,j}V_{j}}{\sum_{j=1}^{M}V_{j}},$$

where k∈{1,2} denotes the normal stress components in the circumferential and radial directions, $\sigma_{kk,j}$ and $V_{j}$ represent the stress and the volume of the $j^{\text{th}}$ element, and M is the total number of elements.

**Figure 11 –:**
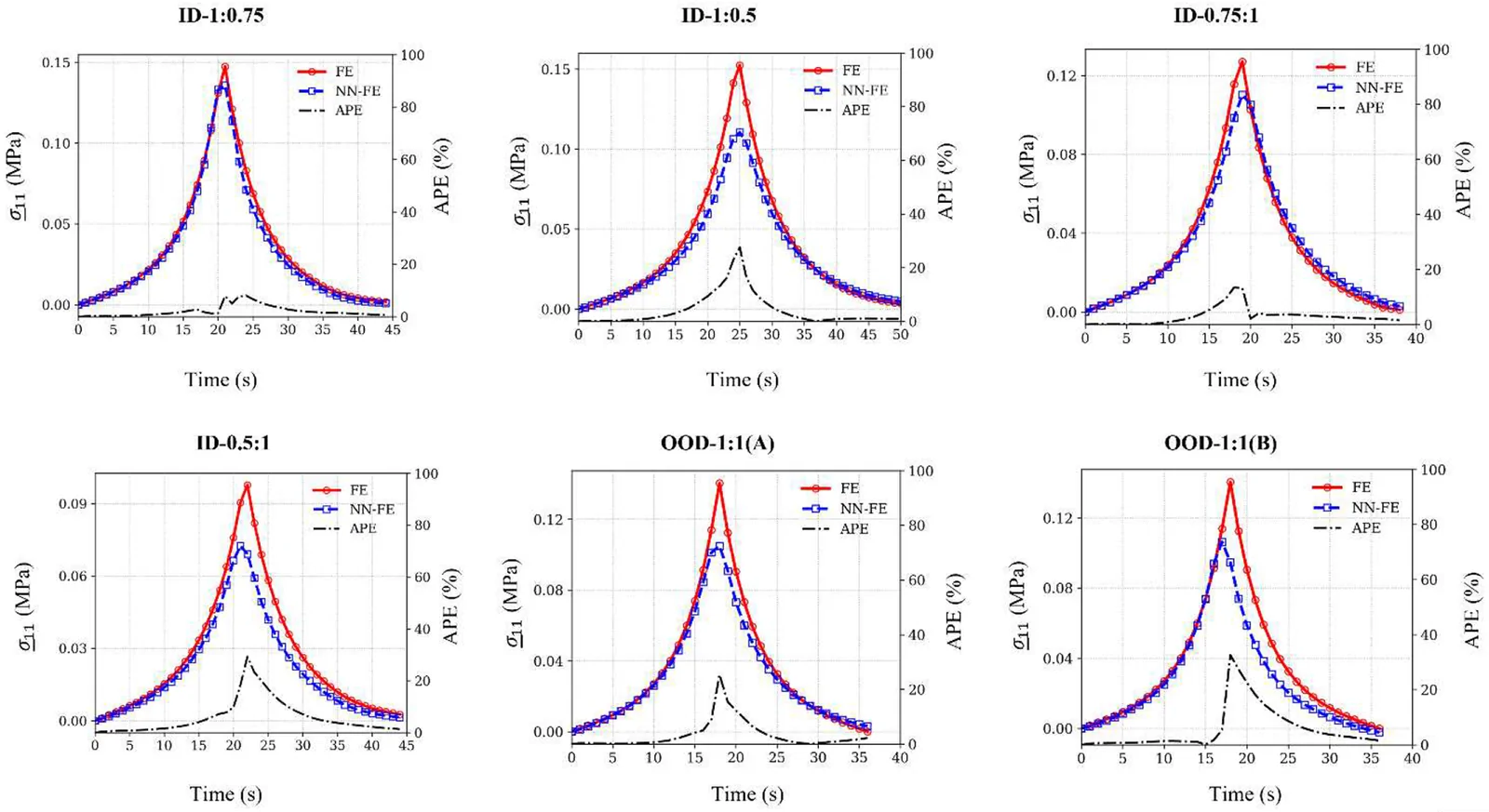
Time-dependent response and volumetric average of the Cauchy normal stress $\left({\underline{\sigma }}_{11}\right)$ for different case studies.

**Figure 12 –:**
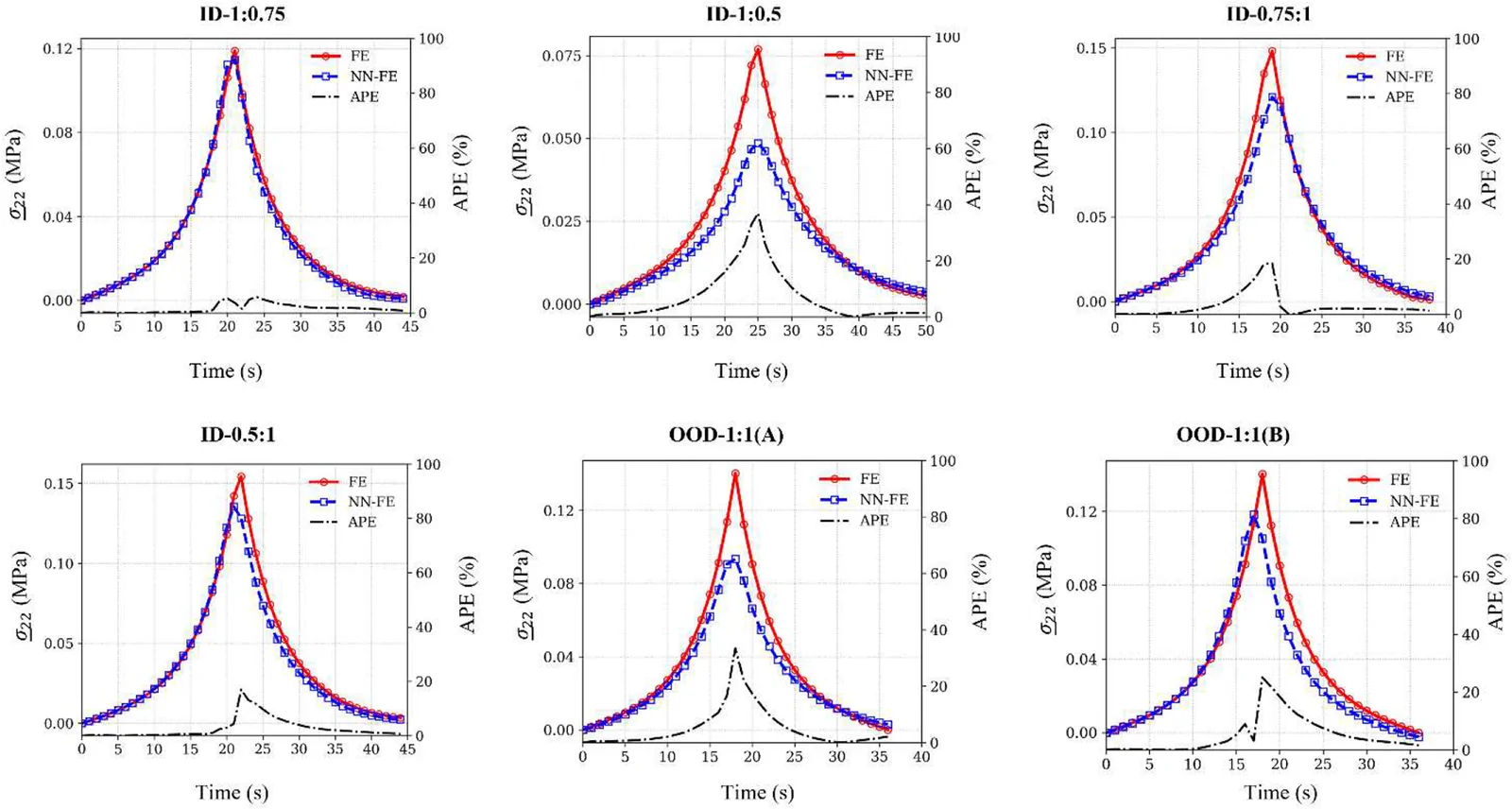
Time-dependent response and volumetric average of the Cauchy normal stress $\left({\underline{\sigma }}_{22}\right)$ for different case studies.

The volume-averaged stress components ${\underline{\sigma }}_{11}$ and ${\underline{\sigma }}_{22}$ predicted by the proposed NN–FE framework are compared with the corresponding FE reference solutions over the full loading cycle, as shown in Figs. 11 and 12, respectively. The black dashed curves indicate APE, referenced to the positive Y-axis. Quantitative metrics including R^2^ and maximum APE values are shown in Table 5.

For the ${\underline{\sigma }}_{11}$ stress component (Fig. 11(a)–(f)), the NN–FE framework demonstrates strong predictive accuracy across most case studies, with $R^{2}$ values ranging from 0.860 (OOD-1:1(A)) to 0.987 (ID-1:0.75). The in-distribution cases ID-1:0.75 and ID-0.75:1 exhibit particularly high fidelity with $R^{2}$ values of 0.987 and 0.975, respectively. The OOD case study OOD-1:1(B) achieves $R^{2}=0.945$, demonstrating robust stress prediction capability for the equibiaxial loading condition. The maximum APE for ${\underline{\sigma }}_{11}$ ranges from 8.05% (ID-1:0.75) to 32.6% (OOD-1:1(A)) across all cases (Table 5).

For the ${\underline{\sigma }}_{22}$ stress component (Fig. 12(a)–(f)), predictive performance remains strong, with $R^{2}$ values spanning 0.813 (ID-1:0.5) to 0.993 (ID-1:0.75). The ID-1:0.75, ID-0.75:1, and ID-0.5:1 cases show particularly high accuracy with $R^{2}$ values exceeding 0.95. The maximum APE for ${\underline{\sigma }}_{22}$ ranges from 5.75% (ID-1:0.75) to 37.0% (ID-1:0.5), as summarized in Table 5.

The temporal stress evolution exhibits similar characteristics to the displacement response. as described in Section 5.3, with peak errors occurring at maximum load. A minor temporal phase lag is observable in certain cases, particularly for Case Studies ID-0.5:1 and the OOD scenarios, reflected in the relatively lower $R^{2}$ values. Despite these temporal lags, the maximum stress prediction errors remain below 37% across all cases, with the majority exhibiting errors <30%.

### 5.5 Stretch ratio and stress field validation

To validate the physical consistency of NN–FE predictions at the local level, stretch ratios and stress fields were evaluated in a central 4×4 element window. Nodal displacements predicted by the NN–FE model were imported into Abaqus to compute a logarithmic strain LE. The principal stretch ratios were obtained from

(26) $$\lambda_{k}=\text{exp}\left(LE_{k}\right),\;k\in \{1,2\},$$

where $LE_{1}$ and $LE_{2}$ represent the logarithmic strains in the X- and Y-directions, respectively. These stretch ratios were used in conjunction with the Fung model to derive the Cauchy stress components $\sigma_{11}$ and $\sigma_{22}$. For comparison purposes, the same procedure was performed using FE displacements to determine the reference solutions.

**Figure 13 –:**
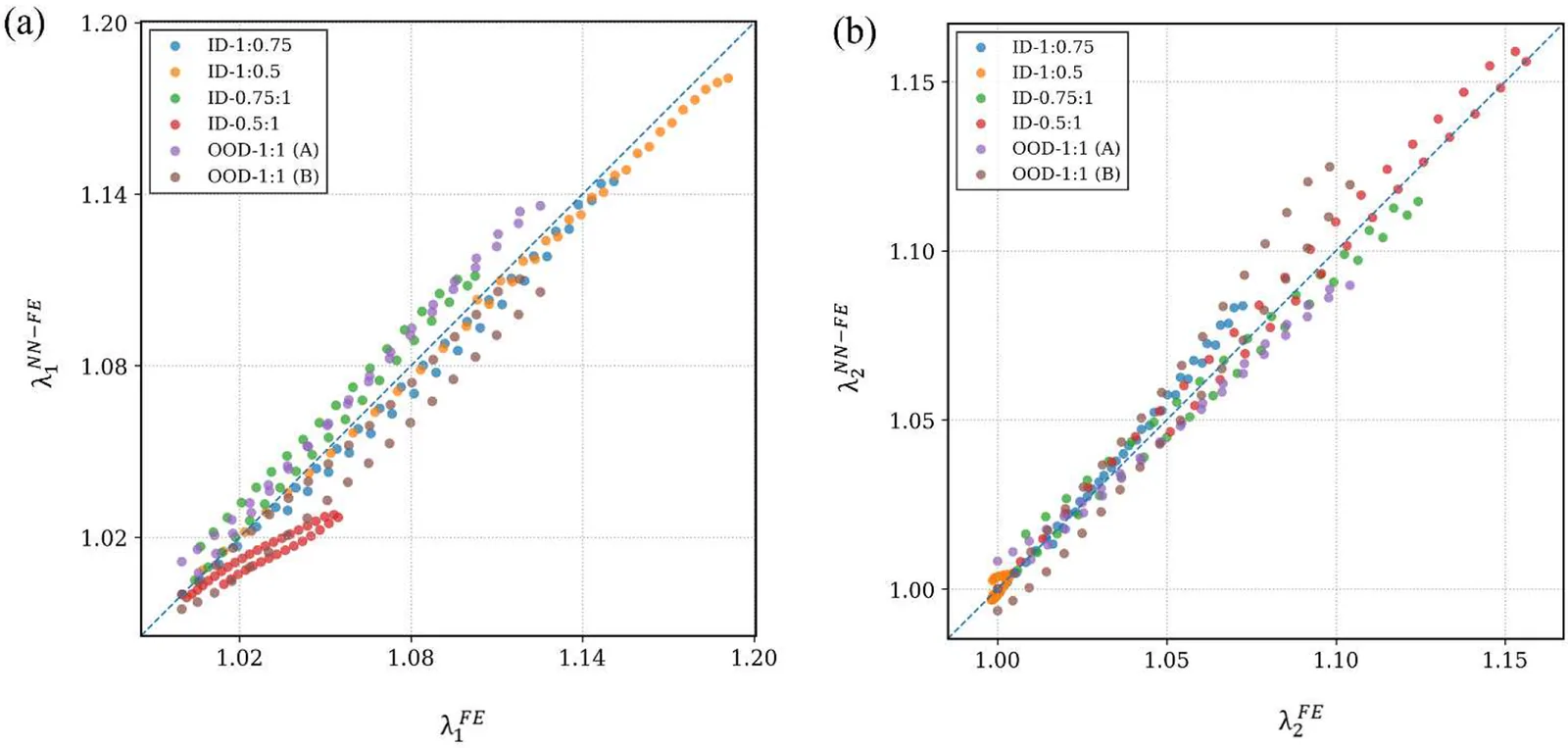
Parity plots comparing the NN–FE predicted stretch ratios against the FE reference solutions for (a) $\lambda_{1}$ and (b) $\lambda_{2}$ across all case studies; data points from all the time steps and loading ratios. The blue dashed line represents perfect agreement (i.e., the identity line).

As shown in Table 6, the RMSE for $\lambda_{2}$ ranges from 0.0050 (ID-1:0.5) to 0.0161 (ID-0.5:1), with MAPE from 0.37% to 1.34% and maximum errors below 2.7%. For $\lambda_{2}$, the RMSE spans 0.0021 to 0.0118, with MAPE below 0.84% and maximum errors under 2.6% (see Table 7). Figure 13 presents parity plots for $\lambda_{1}$ and $\lambda_{2}$, which exhibit strong agreement with the identity line across all case studies and time steps, with minimal scatter.

**Figure 14 –:**
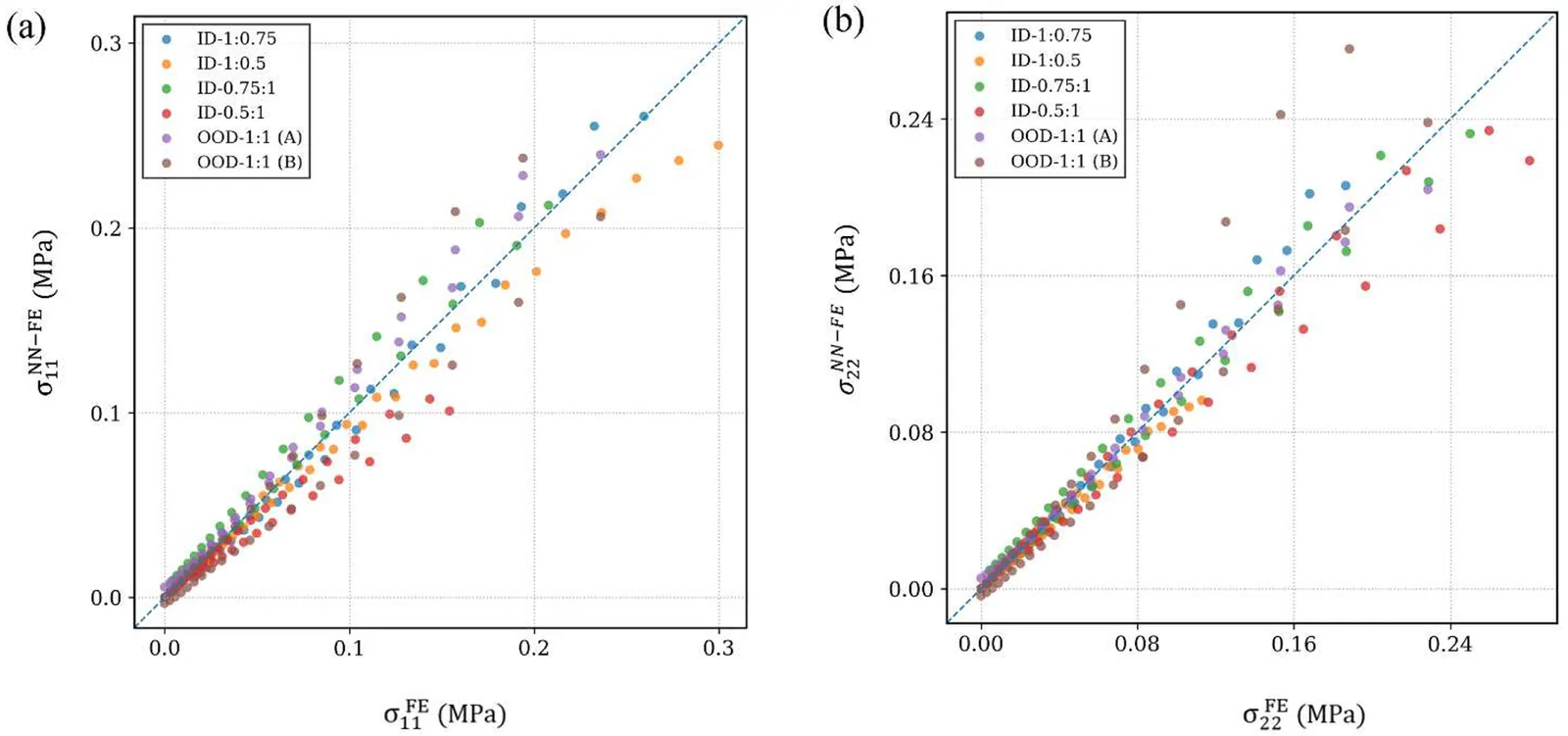
Parity plots comparing the NN–FE predicted normal Cauchy stresses against the FE reference solutions for (a) $\sigma_{11}$ and (b) $\sigma_{22}$ across all case studies; data points from all the time steps and loading ratios. The blue dashed line represents perfect agreement (i.e., the identity line).

For the calculated stress components, $\sigma_{11}$ exhibits an RMSE from 0.0076 to 0.0187 kPa, with MAPE ranging from 2.01% to 7.80% and maximum errors up to 34.6% (Table 8). For $\sigma_{22}$, the RMSE spans from 0.0053 to 0.0257 kPa, with MAPE from 1.40% to 6.33% and maximum errors reaching 38.9% (Table 9). Moreover, Fig. 14 shows parity plots for both stress components, demonstrating good correlation with increased scatter compared to stretch ratios—a result of exponential amplification of the stretch errors through Fung’s model.

**Figure 15 –:**
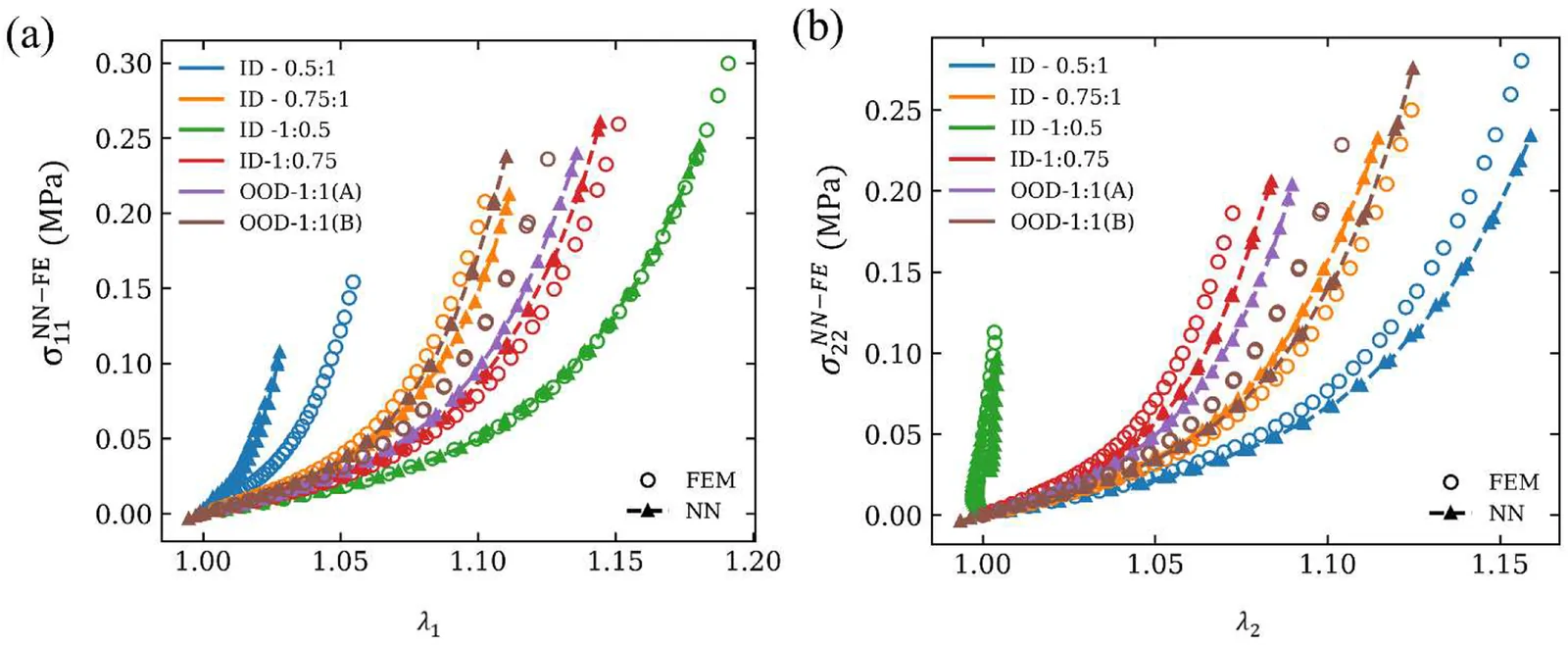
Cauchy stress-stretch relationships comparing the NN-FE predictions (solid triangles) with the FE reference solutions (hollow circles) for (a) $\sigma_{11}-\lambda_{1}$ and (b) $\sigma_{22}-\lambda_{2}$ across all held-out loading ratios.

Finally, Fig. 15 presents stress-stretch relationships comparing NN–FE predictions with FEM reference solutions across all case studies. In general, the proposed framework accurately reproduces the characteristic nonlinear J-shaped response, including the compliant toe region and exponential stiffening. The close overlap between the FE solutions and the NN–FE predictions in most case studies confirms that the framework has learned the underlying constitutive behavior, thereby demonstrating its suitability as a surrogate for conventional FE analysis in characterizing stress-strain behavior.

## 6. Discussion

First, the proposed hybrid NN–FE framework demonstrates robust predictive performance for TVPL mechanics under biaxial loading, with $R^{2}$ values consistently exceeding 0.85 across all six case studies. However, a more detailed analysis reveals three interrelated patterns that shed light on the framework’s performance: (i) temporal phase lag during rapid loading transitions, (ii) systematic error localization at the peak loading and near the constrained boundaries, and (iii) directional sensitivity in prediction accuracy for highly asymmetric loading ratios.

Second, an examination of the time-dependent displacement responses reveals that NN–FE predictions closely track FEM references throughout most of the loading cycle, but a systematic pattern emerges where prediction errors peak at the maximum loading for both $U_{1}$ and $U_{2}$ components across all case studies. This is evident in the APE curves (black dashed curves) that show sharp spikes coinciding with the peak stretch (Figs. 9 and 10). The same temporal pattern is also evident in the calculated stress predictions (Figs. 11 and 12), where the maximum APE—despite the excellent R^2^ values (>0.81 in all cases, see Table 5)—likewise occurs at peak load. This concentration of errors at maximum load is a direct consequence of the Fung model’s exponential stiffening characteristic. Specifically, at high tissue stretches the stress-stretch relationship becomes extremely steep, and small discrepancies in predicted displacement can amplify the errors in the predicted stress. This can be explained as follows: the derivative $\partial \psi /\partial \bar{E}$ in Eq. (6) increases exponentially with strain through the exp(Q) term, causing this error amplification mechanism most pronounced when the exponent Q reaches its maximum at the peak loading.

Additionally, a minor temporal phase lag is observable in several cases, particularly ID-0.5:1 and both OOD scenarios (Figs. 9–12), where the predicted curves slightly lag the reference solutions. This temporal desynchronization occurs primarily during the rapid transitions at the onset and reversal of the loading/unloading patterns, where displacement gradients are largest. Wang et al. [32] demonstrated that PINNs often fail to train effectively due to spectral bias, where neural networks preferentially learn low-frequency components while struggling with high-frequency features and sharp temporal gradients [54–56]. During these rapid transitions, the exponential Fung model response becomes most sensitive to the perturbation, making it challenging for the network to maintain perfect temporal alignment. Nevertheless, the maximum temporal errors remain below 27% for the displacement (Table 4) and 37% for the stress (Table 5), indicating the solver-in-the-loop approach maintains physical consistency and interpretability throughout the loading cycle.

While temporal analysis reveals when errors are largest, the spatial contour plots (Figs. 7 and 8) indicate where they occur. At the peak loading, the maximum absolute percentage errors consistently localize near the specimen edges where displacement boundary conditions are applied through the BioRakes interface. This is evident in the error contours (third column of Figs. 7 and 8) that show concentrated high-error regions at the constrained boundaries while the interior tissue domain exhibits substantially lower errors, typically below 5–10%. Quantitatively, although the maximum localized errors reach 31.0% for $U_{1}$ in Case Study ID-0.5:1 (Table 2) and 36.6% for $U_{2}$ in Case Study ID-1:0.5 (Table 3), the MAPE values across the entire domain remain below 15% and 13%, respectively. This indicates that these peaks are spatially confined to the boundary regions associated with the interplay between kinematic constraints and material response. The kinematic coupling between the reference points and tissue mesh creates localized regions of high displacement gradients, where the neural network’s initial predictions require substantial FE corrections. Studies on biaxial testing of soft tissues have demonstrated that gripping methods and boundary constraints produce significant stress concentrations at specimen edges [57–59], and neural network-based mechanics solvers are known to fail to accurately resolve such concentration features [23]. Furthermore, the anisotropic tissue properties—captured by the direction-dependent parameters in matrix b (see Eq. (17))—lead to a further concentration of deformation along the preferred load paths in the vicinity of these interfaces.

Third, a striking pattern emerges when comparing prediction accuracy across different load ratios that were not considered during model development: model performance deteriorates when the loading ratio used for testing exhibits extreme asymmetry in the direction that was underrepresented during training. For the $U_{1}$ component (Table 2), the Case Study ID-0.5:1, in which the Y-direction load dominates and is twice the load in the X-direction, exhibits a MAPE of 14.9%; this value is significantly higher than that for ID-0.75:1 (2.68%). In contrast, for $U_{2}$ (Table 3), Case Study ID-1:0.5, where the X-directional force dominates, shows a MAPE of 12.2%, far exceeding that of ID-0.75:1 (2.95%). The Study Case ID-0.75:1 achieves the lowest errors for both displacement components, representing a more moderate departure from equibiaxial loading with balanced training coverage of both X-dominant and Y-dominant loading ratios. This directional bias reflects the anisotropic mechanical response of TVPL tissue due to its underlying collagen fiber architecture, fiber recruitment, and fiber reorientation.

**Figure D1 –:**
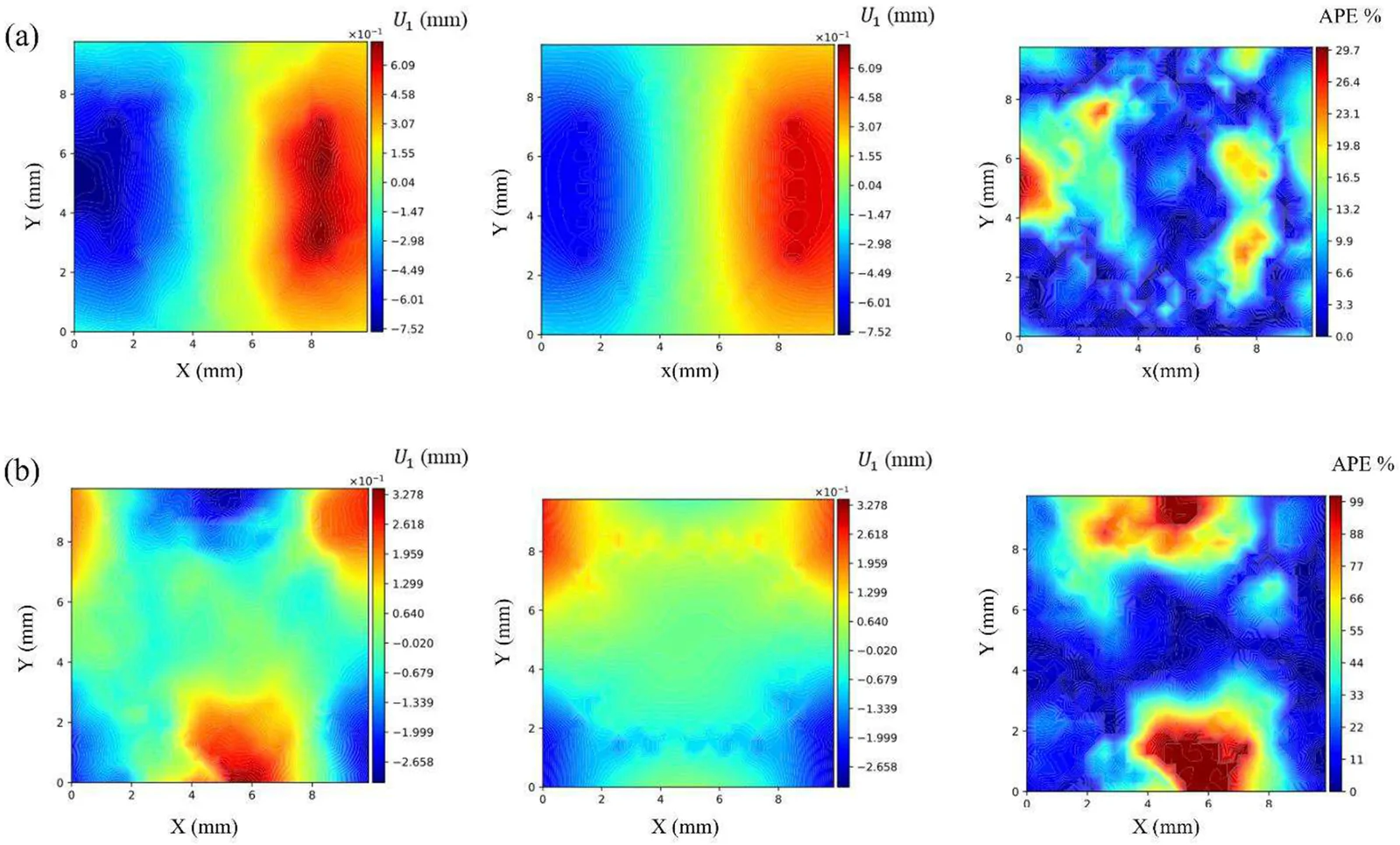
Comparison of displacement contours obtained by the purely data-driven model for case study ID-1:0.5 at peak load. Layout follows Fig. 6: the columns show the predicted field, the FE reference solution, and the absolute percentage error. The rows correspond to: (a) X-displacement component $U_{1}$ and (b) Y-displacement component $U_{2}$.

**Figure D2 –:**
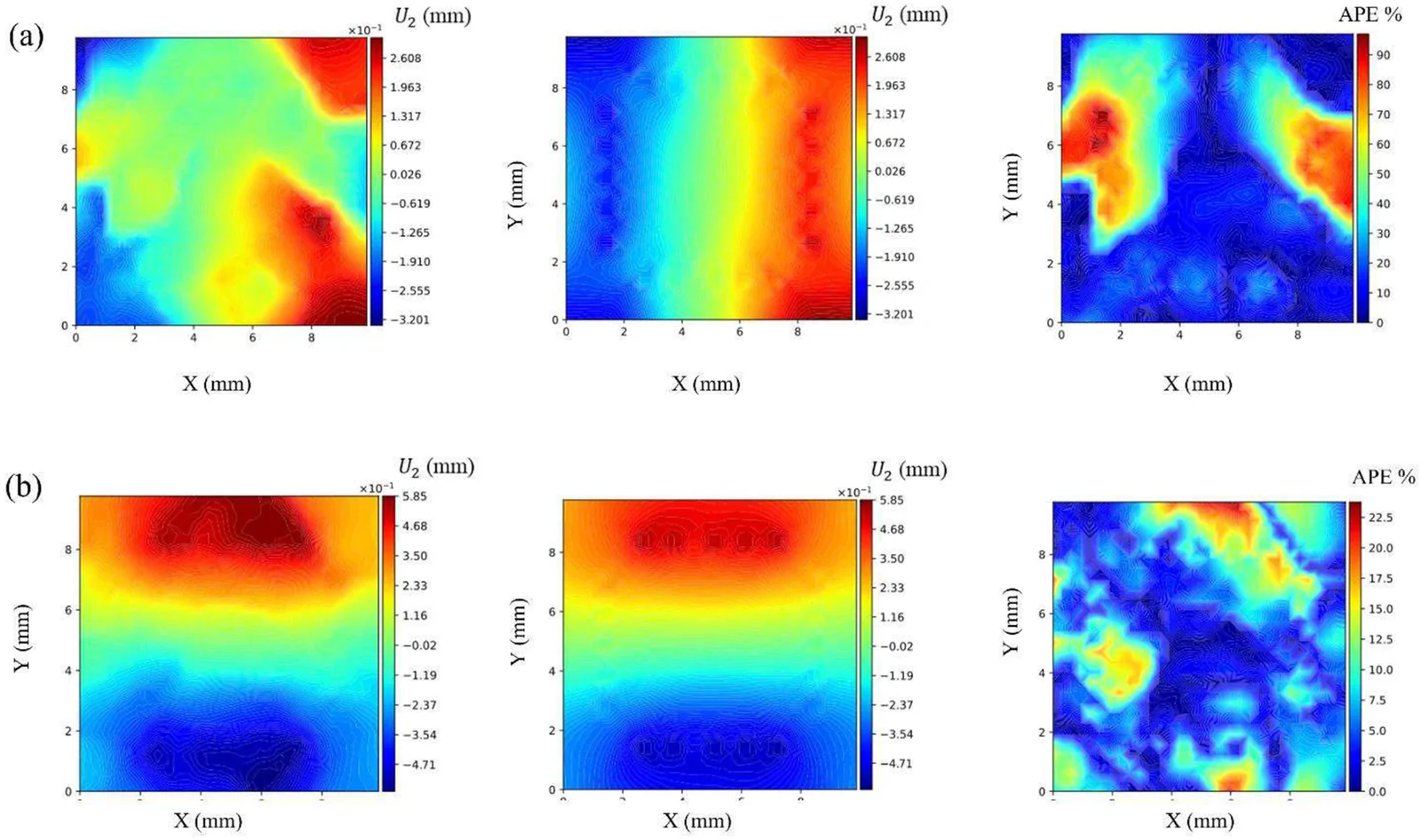
Comparison of displacement contours obtained by the purely data-driven model for case study ID-0.5:1 at peak load. Layout follows Fig. 6: the columns show the predicted field, the FE reference solution, and the absolute percentage error. The rows correspond to: (a) X-displacement component $U_{1}$ and (b) Y-displacement component $U_{2}$.

Prior study on heart valve structure have demonstrated that valve leaflets comprise collagen, elastin, and glycosaminoglycans arranged in directionally oriented microstructures, with biaxial mechanical testing confirming that these tissues exhibit pronounced anisotropy and mechanical properties that vary significantly across different loading directions [27]. The Fung model captures this anisotropy through the coupling terms $b_{1122},b_{1133}$, and $b_{2233}$ (Eq. (9)), which govern how loading in one direction influences the perpendicular response. When testing on extreme asymmetric ratios such as $F_{x}:F_{y}=0.5:1$ or $F_{x}:F_{y}=1:0.5$, the neural network must extrapolate into regions of the coupled stress-strain space that were sparsely sampled during training. Remarkably, these in-distribution cases with extreme directional asymmetry can perform even worse than the equibiaxial out-of-distribution scenarios. For the $U_{1}$ component, Case Study ID-0.5:1 exhibits a MAPE of 14.9% and the maximum error of 31.0%, both substantially higher than those in OOD-1:1(A): i.e., a 2.74% MAPE and 15.2% maximum error, and greater than those in OOD-1:1(B): 9.60% MAPE and 24.5% maximum error. Similarly, for $U_{2}$, Case Study ID-1:0.5 shows a MAPE of 12.2% and the maximum error of 36.6%, higher than those in OOD-1:1(A): 8.17% MAPE and 27.1% maximum error, and noticeably higher than those in OOD-1:1(B): 3.36% MAPE and 14.4% maximum error. This counterintuitive result underscores that the challenge lies not merely in the load situation itself, but rather in the degree of directional imbalance relative to the training distribution; in this context, extremely asymmetric conditions can lead to prediction difficulties that ga beyond those encountered with balanced load situations—even when the latter lie entirely outside the training dataset. Nevertheless, our NN–FE model outperformed the purely data-driven model with empirical learning that fails to maintain essential physical consistency and interpretability under extreme biaxial loading (see more details in Appendix D and Figs. D1–D2).

Finally, despite the identified error patterns the proposed NN–FE framework demonstrates several strengths that demonstrate its viability as a computational surrogate. The stretch ratio predictions maintain exceptional accuracy with the MAPE <1.4% (Tables 6 and 7) and a strong agreement with the FE reference solutions, as shown in the parity plots (Fig. 13). The stress predictions, though exhibiting larger relative errors due to the exponential amplification through the constitutive relationship, remain acceptable (i.e., MAPE<7.8%, see Tables 8 and 9) and accurately reproduce the characteristic J-shaped stress-stretch curves throughout loading and unloading phases (Fig. 15). The close alignment between NN–FE predictions and FE ground-truth across all loading ratios confirms that the proposed framework has learned the underlying constitutive behavior of Fung’s model, capturing both the compliant toe region at lower stretches and the exponential stiffening at higher stretches. This physical consistency, combined with better computational efficiency regarding the inference time in comparison to the traditional full-scale FE analysis, makes the framework suitable for applications requiring rapid mechanical predictions, such as real-time surgical planning or iterative device design optimization. The consistent training convergence (Fig. 6), where all case studies stabilize within approximately 2 × 10^4^ epochs without divergence, demonstrates the robustness of the solver-in-the-loop training strategy.

## 7. Conclusion and Future Perspectives

In this study, we presented a hybrid, physics-informed neural network framework that combines a feedforward neural network with a nonlinear FE solver to predict the anisotropic hyperelastic response of a representative cardiovascular tissue under quasi-static biaxial loading. The framework employs a solver-in-the-loop approach where neural network predictions are refined through FE analysis to ensure satisfaction of mechanical equilibrium. Unlike the conventional PINNs, this design can prevent convergence issues in strongly nonlinear hyperelastic problems, while maintaining computational efficiency.

Moreover, we have validated the framework using leave-one-ratio-out cross-validation across six case studies. The model achieved a MAPE <15% for the displacement predictions, with temporal correlation $R^{2}$ reaching 0.997 for in-distribution cases and >0.85 for out-of-distribution scenarios. Stretch ratio validation yielded a MAPE <1.4%, with the stress predictions maintaining a MAPE <7.8%. It has been shown that the proposed framework successfully reproduces the characteristic J-shaped stress-stretch curves across all loading and unloading phases, indicating successful learning of the tissue’s constitutive behavior. We have found that the concentration of errors near boundaries is attributable to the solver-in-the-loop architecture and the exponential sensitivity of Fung’s model to displacement gradient variations. Despite these localized boundary effects, we have demonstrated that the framework successfully reproduces overall deformation patterns, with predicted contours closely matching the FE reference fields throughout the interior domain.

Although the present framework demonstrates high accuracy and efficiency for quasi-static hyperelastic models, limitations remain that must be addressed to enhance practical applicability. First, the current workflow is restricted to quasi-static loading conditions; extending the framework to dynamic physiological environments and rate-dependent phenomena, such as tissue viscoelasticity, remains an open challenge. Second, current validation relies on a single tissue specimen, which limits the immediate generalizability of the trained network parameters given the inherent biological variability. Future work must explore the applicability of this approach to patient-specific geometries, heterogeneous material properties, and spatially varying fiber orientations within larger cohorts comprising multiple samples.

Finally, when generalizing to unseen data, the model exhibits locally high errors in the boundary regions. This discrepancy is fundamentally driven by the highly nonlinear, exponential behavior of the tissue, which changes significantly across different loading directions. Because our experimental protocol was restricted to seven loading ratios, the network had a relatively sparse sampling of the highly coupled anisotropic stress-strain space. For high-precision clinical applications such as real-time surgical planning, resolving these boundary features accurately is paramount. Consequently, future research should implement more comprehensive testing protocols with a higher density of loading ratios to better capture the tissue’s full mechanical spectrum; furthermore, advanced adaptive training strategies or refined weighting of loss functions should be investigated to smooth gradients at the interface.

To conclude, this study represents an important first step toward efficient surrogate modeling of cardiac valve mechanics and lays the foundation for future advances in real-time biomechanical simulations. This work highlights the immense potential of combining FE methods and PINNs to address challenging, highly nonlinear problems in cardiovascular biomechanics.

## Figures and Tables

**Table 1 –:** Fung model parameters estimated by fitting to biaxial data for all seven $F_{x}:F_{y}$ loading ratios.

Parameter	c[MPa]	κ[MPa]	$b_{1111}$	$b_{2222}$	$b_{3333}$	$b_{1122}$	$b_{1133}$	$b_{2233}$
Value	0.0063	0.055	23.214	23.834	34.879	1.434	2.099	0.0021

**Table 2 –:** Comparison of the NN–FE predicted X-displacement field $U_{1}$ against the FE reference solution at peak loading across all case studies. ID = in-distribution; OOD = out-of-distribution.

Cases	Root Mean Square Error (mm)	Mean Absolute Percentage Error	Maximum Absolute Percentage Error
ID-1:0.75	0.0083	1.24%	6.81%
ID-1:0.5	0.0165	2.14%	8.62%
ID-0.75:1	0.0140	2.68%	11.01%
ID-0.5:1	0.0511	14.91%	30.98%
OOD-1:1(A)	0.0156	2.74%	15.18%
OOD-1:1(B)	0.0517	9.60%	24.53%

**Table 3 –:** Comparison of the NN–FE predicted Y-displacement field $U_{2}$ against the FE reference solution at peak loading across all case studies. ID = in-distribution; OOD = out-of-distribution.

Cases	Root Mean Square Error (mm)	Mean Absolute Percentage Error	Maximum Absolute Percentage Error
ID-1:0.75	0.0179	5.00%	16.35%
ID-1:0.5	0.0408	12.22%	36.59%
ID-0.75:1	0.0159	2.95%	10.68%
ID-0.5:1	0.0154	2.44%	8.62%
OOD-1:1(A)	0.0395	8.17%	27.12%
OOD-1:1(B)	0.0173	3.36%	14.36%

**Table 4 –:** Coefficient of determination $R^{2}$ and maximum absolute percentage error (APE) between the NN-FE and FE solutions for the time-dependent root mean square (RMS) displacements $U_{1}$ and $U_{2}$ across all held-out in-distribution (ID) and out-of-distribution (OOD) case studies.

Cases	$RMS\left(U_{1}\right)\;R^{2}$	$RMS\left(U_{2}\right)\;R^{2}$	$RMS\left(U_{1}\right)$ Max. APE	$RMS\left(U_{2}\right)$ Max. APE
ID-1:0.75	0.988	0.977	5.4%	9.8%
ID-1:0.5	0.997	0.835	3.9%	24.0%
ID-0.75:1	0.954	0.987	10.1%	6.4%
ID-0.5:1	0.725	0.982	26.5%	8.1%
OOD-1:1(A)	0.984	0.954	9.4%	14.2%
OOD-1:1(B)	0.892	0.956	16.6%	10.5%

**Table 5 –:** Coefficient of determination $R^{2}$ and maximum absolute percentage error (APE) between the NN-FE and FE solutions for the volume-averaged Cauchy stresses ${\underline{\sigma }}_{11}$ and ${\underline{\sigma }}_{22}$ across all held-out in-distribution (ID) and out-of-distribution (OOD) case studies.

Cases	${\underline{\sigma }}_{11}{\;R}^{2}$	${\underline{\sigma }}_{22}{\;R}^{2}$	${\underline{\sigma }}_{11}$ Max. APE	${\underline{\sigma }}_{22}$ Max. APE
ID-1:0.75	0.987	0.993	8.1%	5.8%
ID-1:0.5	0.922	0.813	27.6%	37.0%
ID-0.75:1	0.975	0.959	13.4%	18.4%
ID-0.5:1	0.891	0.970	29.3%	16.9%
OOD-1:1(A)	0.945	0.884	25.3%	33.4%
OOD-1:1(B)	0.860	0.904	32.6%	25.0%

**Table 6 –:** Comparison of the stretch ratio $\lambda_{1}$ in the X-direction derived from the NN–FE model against the FE reference solution at the central tissue region across all case studies.

Cases	Root Mean Square Error (−)	Mean Absolute Percentage Error	Maximum Absolute Percentage Error
ID-1:0.75	0.0066	0.50%	0.97%
ID-1:0.5	0.0050	0.37%	0.88%
ID-0.75:1	0.0094	0.73%	1.37%
ID-0.5:1	0.0161	1.34%	2.61%
OOD-1:1(A)	0.0101	0.84%	1.41%
OOD-1:1(B)	0.0126	0.93%	1.77%

**Table 7 –:** Comparison of the stretch ratio $\lambda_{2}$ in the Y-direction derived from the NN-FE model against the FE reference solution at the central tissue region across all case studies.

Cases	Root Mean Square Error (−)	Mean Absolute Percentage Error	Maximum Absolute Percentage Error
ID-1:0.75	0.0057	0.40%	1.24%
ID-1:0.5	0.0021	0.17%	0.42%
ID-0.75:1	0.0055	0.41%	0.94%
ID-0.5:1	0.0052	0.37%	0.80%
OOD-1:1(A)	0.0065	0.50%	1.30%
OOD-1:1(B)	0.0118	0.84%	2.59%

**Table 8 –:** Comparison of the normal Cauchy stress $\sigma_{11}$ in the X-direction derived from the NN–FE predicted stretch ratios against the FE reference solution across all case studies.

Cases	Root Mean Square Error (MPa)	Mean Absolute Percentage Error	Maximum Absolute Percentage Error
ID-1:0.75	0.0076	2.01%	8.69%
ID-1:0.5	0.0162	3.46%	18.3%
ID-0.75:1	0.0113	3.27%	15.6%
ID-0.5:1	0.0180	7.80%	34.6%
OOD-1:1(A)	0.0111	3.30%	14.6%
OOD-1:1(B)	0.0187	5.46%	21.8%

**Table 9 –:** Comparison of the normal Cauchy stress $\sigma_{22}$ in the Y-direction derived from the NN–FE predicted stretch ratios against the FE reference solution across all case studies.

Cases	Root Mean Square Error (MPa)	Mean Absolute Percentage Error	Maximum Absolute Percentage Error
ID-1:0.75	0.0092	2.75%	18.1%
ID-1:0.5	0.0053	3.25%	14.8%
ID-0.75:1	0.0092	2.89%	8.42%
ID-0.5:1	0.0181	3.67%	22.0%
OOD-1:1(A)	0.0055	1.40%	10.7%
OOD-1:1(B)	0.0257	6.33%	38.9%

**Figure A1 –:**
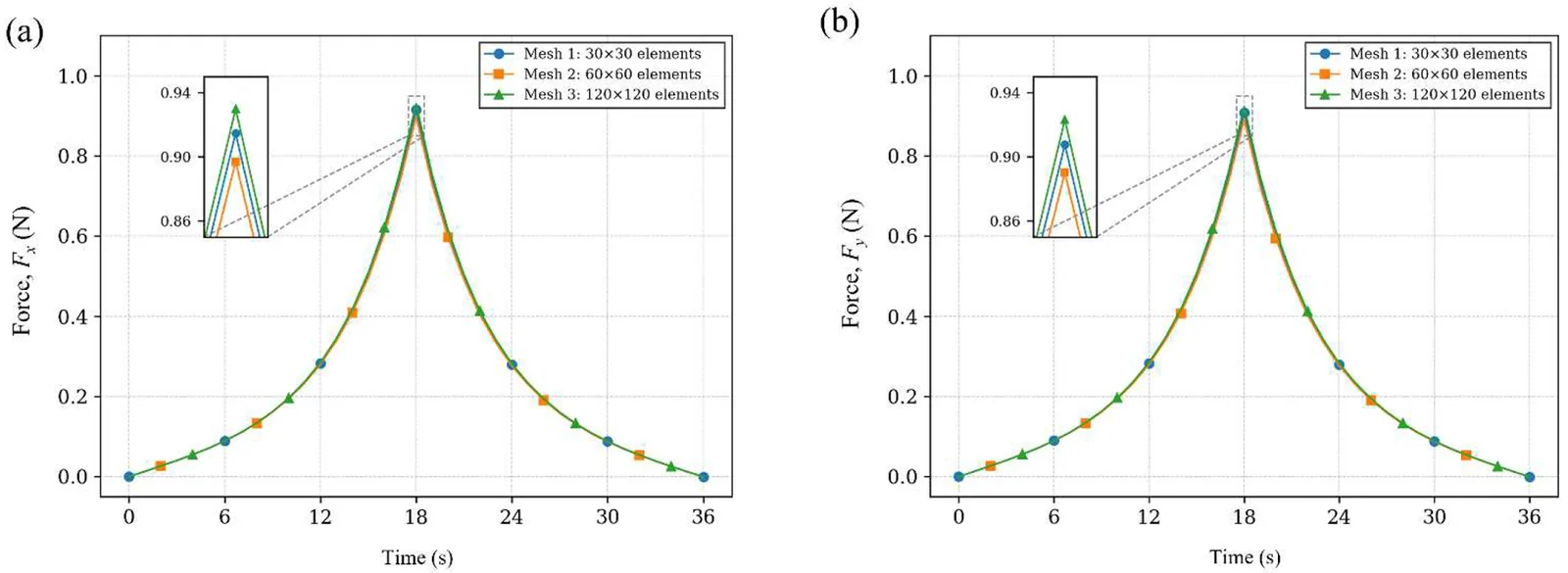
Comparison of simulated reaction force vs time histories for three uniform meshes successively refined in the (a) X-direction and (b) Y-direction. The curves represent Mesh 1 (30 × 30 elements), Mesh 2 (60 × 60 elements), and Mesh 3 (120 × 120 elements), with magnified details highlighting the close convergence of forces near the peak loading at t=18s for the 1:1 loading ratio.

**Figure B1 –:**
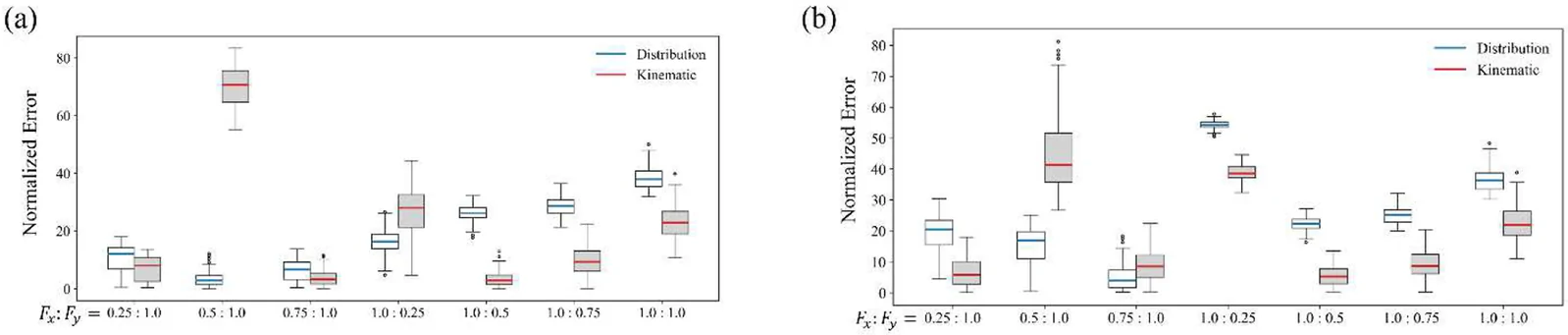
Comparison of the absolute percentage error (normalized by peak experimental force) between the simulated reference point (RP) reaction forces and the experimental load-cell force measurements for (a) the X-direction and (b) the Y-direction considering two coupling methods: (i) kinematic coupling and (ii) distribution coupling. The box plots represent the error distributions across all time steps and seven loading ratios.

**Figure B2 –:**
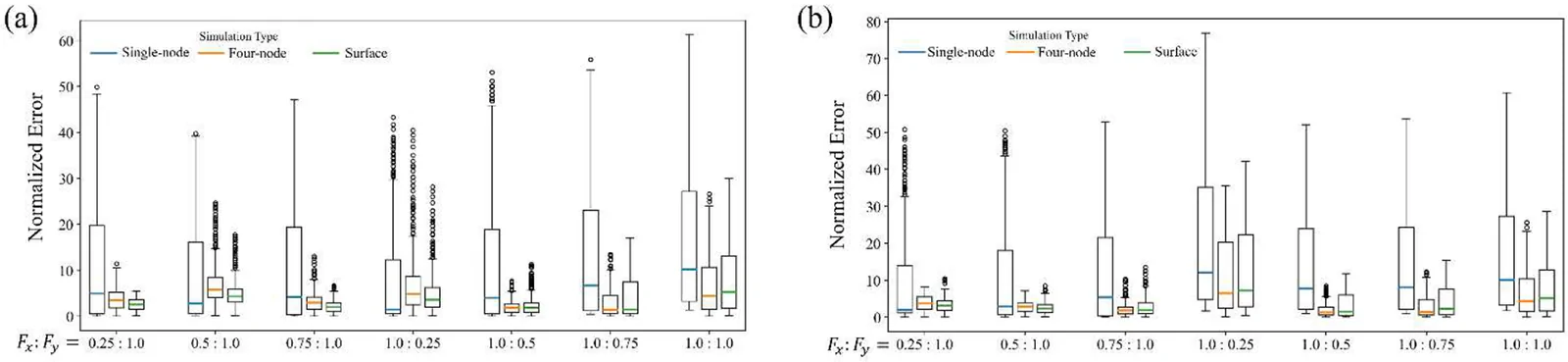
Comparison of the absolute percentage error (normalized by peak experimental force) between the simulated forces and the experimental load-cell force measurements for (a) the X-direction and (b) the Y-direction across three connection strategies: (i) single-node, (ii) four-nodes, and (iii) surface-based attachment. The box plots represent the error distributions across all time steps and seven loading ratios.

## Appendix A: Mesh Convergence Study

To determine the optimal mesh density for the FE simulations, a mesh convergence study was performed using three successively refined uniform meshes: i.e., Mesh 1: 30 × 30 elements, Mesh 2: 60 × 60 elements, and Mesh 3: 120 × 120 elements. The reaction forces in the X- and Y-directions $\left(F_{X},F_{Y}\right)$ were monitored over the full loading cycle for the 1:1 loading ratio. As shown in Fig. A1, all three grids yield nearly identical force–time histories, with differences becoming visible only near the peak load at t=18s. At this peak, the $F_{X}$ values were 0.915, 0.897, and 0.930 N for Meshes 1, 2, and 3, respectively, while the corresponding $F_{Y}$ values were 0.908, 0.890, and 0.923 N. The relative difference in peak values $F_{X}$ between Mesh 1 and Mesh 3 is approximately 1.6%, and approximately 1.7% for $F_{Y}$; this demonstrates that even the coarsest mesh captures the structural response with sufficient accuracy. Since Mesh 1 (30 × 30 elements) yields results consistent with those of the finer meshes while requiring the least computational cost—a crucial factor for the iterative NN–FE training loop—it was selected as the mesh configuration for all subsequent FE simulations in this study.

## Appendix B: BioRake-Tissue Interface Connection in Abaqus Simulation

To determine the optimal connection strategy between the reference points (RPs) and the tissue mesh in Abaqus, parametric studies were conducted to evaluate various coupling configurations. In these studies, the predicted reaction forces at the RPs throughout the entire loading cycle— and for all seven load ratios—were compared with experimental force measurements obtained from load cells. Subsequently, the error was quantified as the absolute difference between the simulated and experimental forces at each time step and normalized by the respective peak force along each corresponding axis.

First, two coupling types were considered: (i) kinematic coupling and (i) distributing coupling. Figure B1 shows that, except for a few loading ratios, kinematic coupling achieved consistently lower median errors and tighter error distributions compared to distributing coupling for both force directions, suggesting that kinematic coupling is better in resolving the reaction forces and thus was selected for further evaluation.

Next, three connection strategies were then evaluated via kinematic coupling: (i) single-node, (ii) four-node, and (iii) surface-based attachment. Figure B2 shows that the four-node connection consistently exhibited the lowest median error and reduced variability compared to single-node and surface-based attachment methods. The connection via a single node exhibited the largest dispersion in prediction errors, particularly under asymmetric loading ratios, with extreme errors in the X-direction reaching up to 50%. Based on these results, kinematic coupling with four-node attachment was selected for all subsequent simulations.

## Appendix C: Performance Metrics and Evaluation Criteria

We consider the following metrics for all quantitative comparisons between the NN-FE predictions and FE reference solutions:

### (i) Root Mean Square Error (RMSE)

For a given field quantity ϕ (i.e., displacement, stretch ratio, or Cauchy stress), the RMSE is computed as

(C. 1)
$$\text{RMSE}(\phi )=\sqrt{\frac{1}{N}\sum_{i=1}^{N}{\left(\phi_{i}^{\text{NN}-\text{FE}}-\phi_{i}^{\text{FE}}\right)}^{2}},$$

where N is the number of evaluation points (e.g., nodes for displacement fields, elements for stress fields, or time steps for temporal comparisons), $\phi_{i}^{\text{NN}-\text{FE}}$ represents the NN–FE predicted value, and $\phi_{i}^{\text{FE}}$ denotes the FE reference solution obtained from Abaqus.

### (ii) Mean Absolute Percentage Error (MAPE)

The MAPE quantifies the average relative percentage error as

(C. 2)
$$\text{MAPE}(\phi )=\frac{1}{N}\sum_{i=1}^{N}\frac{\left|\phi_{i}^{\text{NN}-\text{FE}}-\phi_{i}^{\text{FE}}\right|}{\left|\phi_{i}^{\text{FE}}\right|}100\%.$$

For temporal stress comparisons, where instantaneous values may approach zero during unloading, the MAPE is computed only over the loading phases where $\left|\phi_{i}^{\text{FE}}\right|>0.01\;\text{max}\left(\left|\phi^{\text{FE}}\right|\right)$.

### (iii) Coefficient of Determination $\left(R^{2}\right)$

For time-dependent quantities, the temporal correlation is assessed using

(C. 3)
$$R^{2}=1-\frac{\sum_{i=1}^{N_{t}}{\left(\phi_{i}^{\text{NN}-\text{FE}}-\phi_{i}^{\text{FE}}\right)}^{2}}{\sum_{i=1}^{N_{t}}{\left(\phi_{i}^{\text{NN}-\text{FE}}-{\underline{\phi }}^{\text{FE}}\right)}^{2}},$$

where $N_{t}$ is the number of time steps in the loading cycle and ${\underline{\phi }}^{\text{FE}}$ denotes the temporal average of the FE reference solution.

### (iv) Maximum Absolute Percentage Error (max. APE)

The maximum pointwise relative error is defined as

(C. 4)
$$\text{max}\;APE(\phi )=\text{max}\left(\frac{\left|\phi_{i}^{\text{NN}-\text{FE}}-\phi_{i}^{\text{FE}}\right|}{\left|\phi_{i}^{\text{FE}}\right|}\right)100\%.$$

This metric identifies the worst-case local discrepancy, typically occurring at the peak loading or near the kinematic-constrained boundaries.

## Appendix D: Baseline Comparison for Pure Data-Driven Modeling

To contextualize the performance of the hybrid NN–FE framework on extreme asymmetric loading ratios, a purely data-driven baseline was established. A neural network with identical architecture (i.e., six hidden layers, 128 neurons per layer, tanh activation) was trained directly on FE displacement data without solver-in-the-loop integration. The mean squared error between the predicted and target nodal displacements was minimized using the neural network, employing the same ADAM optimizer and a cosine annealing schedule. The training data included all loading ratios except for the case reserved for validation, in accordance with the leave-one-ratio-out protocol of the hybrid NN-FE framework.

The spatial displacement contours at peak loading for the two most challenging cases identified in the main text (i.e., ID-1:0.5 and ID-0.5:1) are presented in Figs. D1 and D2, respectively. For case study ID-1:0.5, the data-driven model exhibits the maximum errors of 29.7% for $U_{1}$ and 99% for $U_{2}$. For case study ID-0.5:1, the maximum errors reach 90% for $U_{1}$ and 22.5% for $U_{2}$. In contrast, the proposed hybrid NN–FE framework achieves substantially lower maximum errors: i.e., 8.6% $\left(U_{1}\right)$ and 36.6% $\left(U_{2}\right)$ for case study ID-1:0.5, and 31.0% $\left(U_{1}\right)$ and 8.6% $\left(U_{2}\right)$ for case study ID-0.5:1 (see also Tables 2 and 3).

Overall, the purely data-driven approach fails to maintain essential physical consistency under extreme biaxial loading; errors are distributed across the entire domain rather than remaining confined to the boundaries. This comparison demonstrates that integrating a solver is essential for generalizing the learned NN–FE model to asymmetric loading conditions; under such conditions, purely empirical learning reaches its limits in capturing the coupled anisotropic response described by the constitutive Fung model.
